# Systematic mapping of small nucleolar RNA interactions in human cells

**DOI:** 10.1080/15476286.2025.2589573

**Published:** 2025-11-14

**Authors:** Hywel Dunn-Davies, Tatiana Dudnakova, Jean-Louis Langhendries, Nicholas Watkins, Denis L.J. Lafontaine, David Tollervey

**Affiliations:** a Centre for Cell Biology, School of Biological Sciences, University of Edinburgh, Edinburgh, UK; b RNA Molecular Biology, Fonds de la Recherche Scientifique (F.R.S./FNRS), Université Libre de Bruxelles (ULB), Gosselies, Belgium; c Institute for Cell and Molecular Biosciences, Newcastle University, Newcastle Upon Tyne, UK

**Keywords:** Small nucleolar RNA, snoRNA, RNA-RNA interaction, RNA-protein interaction, UV cross-linking, methylation, RNA modification, epitranscriptomics

## Abstract

Altered expression of box C/D small nucleolar RNAs (snoRNAs) is implicated in human diseases, including cancer. Box C/D snoRNAs canonically direct site-specific, 2’-*O*-methylation but the extent to which they participate in other functions remains unclear. To identify RNA interactions of box C/D snoRNAs in human cells, we applied two techniques based on UV crosslinking, proximity ligation and sequencing of RNA hybrids (CLASH and FLASH). These identified hundreds of novel snoRNA interactions with rRNA, snoRNAs and mRNAs. We developed an informatic pipeline to rigorously call interactions predicted to direct methylation. Multiple snoRNA-rRNA interactions identified were not predicted to direct RNA methylation. These potentially modulate methylation efficiency and/or contribute to folding dynamics during ribosomal subunit biogenesis. snoRNA-mRNA hybrids included 1,300 interactions between 117 snoRNA families and 940 mRNAs. Human U3 is substantially more abundant than other snoRNAs and represented about 50% of snoRNA-mRNA hybrids. The distribution of U3 interactions across mRNAs also differed from other snoRNAs. Following U3 depletion, mRNAs showing altered abundance were strongly enriched for U3 CLASH interactions. Most human snoRNAs are excised from pre-mRNA introns. Enrichment for snoRNA association with branch point regions of introns that contain snoRNA genes was common, suggesting widespread regulation of snoRNA maturation.

## KEY POINTS

- Dataset for human snoRNA interactions
- Pipeline for calling methylation guides
- rRNA modification sites are targeted by competing, non-guide snoRNAs
- Splicing signals in introns that encode snoRNAs are preferentially targeted for snoRNA binding

## Introduction

The small nucleolar RNAs (snoRNAs) are a class of abundant, small stable RNAs, most of which act as guides for site-specific RNA modification [1]. Most members of the box C/D class of snoRNAs select sites of ribose 2’-*O*-methylation by extended regions of perfect complementarity with target sites (≥12 bp), in which the nucleotide to be modified is placed exactly 5 bp from the conserved box D or box D’ motifs within the snoRNA (reviewed in [2,3]). The box C/D snoRNAs associate with a group of four common proteins, NOP56, NOP58, 15.5K and the methyltransferase Fibrillarin (FBL). The snoRNAs have a partially symmetrical structure, in which stem structures bring together the highly conserved, terminal box C (RUGAUGA, *R* = A or G) and box D (CUGA) sequences and the related, but less conserved, internal box C’ and box D’ elements. These stem structures include a K-turn structural motif that is bound by the small 15.5K protein. *In vitro* structural analysis indicated that the box C/D stem is also bound by NOP58, while the box C’/D’ stem is bound by the homologous NOP56 protein. Each region is bound by a copy of FBL, so the regions flanking either box D, box D’ or both can function as methylation guides. In human cells, about a third of the ~110 snoRNA-directed methylation sites show variable stoichiometry depending on physiological contexts, indicating regulation that is likely to be functionally important (reviewed in [4–6]).

The strict requirement for a long region of perfect complementarity that extends to box D/D’ for guide function implies that strong snoRNA base pairing could occur without eliciting target RNA methylation. Indeed, a small number of box C/D snoRNAs have essential functions in ribosome synthesis that require snoRNA/pre-rRNA base pairing without associated rRNA methylation. In vertebrates, these snoRNAs include U3, U14 and U8 required for processing (reviewed in [3]). In addition, pre-rRNA base-pairing by U13 snoRNA directs formation of *N*^4^-acetyl cytidine (ac4C) by NAT10, rather than methylation [7,8].

Other acetylation targets are not known, but human snoRNAs can direct methylation of small RNAs, including spliceosomal small nuclear RNAs (snRNAs) and other snoRNAs [9,10]. A number of human snoRNAs have been reported to bind mRNAs, with diverse effects including pre-mRNA splicing, 3’ end formation and protein secretion [11–14]. Human snoRNAs show tissue-specific expression patterns, with altered expression linked to disease and tumorigenesis [15–22]. The imprinted, brain-specific snoRNAs snoRD115 and snoRD116 are implicated in the neurological disease Prader-Willi syndrome (see [12,23–26] reviewed in [27]). Mutations in U8/snoRD118 cause the neurological disease leukoencephalopathy with calcification and cysts in humans [28] and a Zebrafish model [29]. In addition, snoRNAs can apparently function by direct protein binding: Loss of specific snoRNAs reduced levels of the GTP-bound, active form of K-Ras with consequent hyperactivation of the Ras-ERK1/ERK2 signalling pathway [18]. snoRNAs were also implicated in activation of the immune regulator Protein Kinase RNA-activated (PKR) under conditions of metabolic stress [30].

Bioinformatics approaches have been used to predict snoRNA binding sites in several systems, particularly where this is associated with methylation [31–35]. In addition, a number of recent reports have described methods for the identification of snoRNA-target RNA interactions through proximity ligation followed by sequencing of the products of reverse transcription and PCR amplification (RT-PCR) [36–41]. The crosslinking and sequencing of hybrids (CLASH) approach uses stringent tandem affinity purification including denaturing conditions to recover RNA-protein and RNA-RNA interactions involving yeast snoRNAs [37,38] and human miRNAs [42,43]. Previous analyses in HEK cells established that CLASH predominately faithfully recovered *in vivo* interactions, rather than *in vitro* contact after lysis [42]. A related approach, formaldehyde-assisted crosslinking and sequencing of hybrids (FLASH) combines immunoprecipitation with mild chemical crosslinking, which stabilizes protein complexes allowing denaturing wash conditions [36].

Here we report the use of CLASH and FLASH to systematically map the interactions between box C/D snoRNAs and the human transcriptome.

## Results

### Systematic mapping of box C/D snoRNA interactions by UV crosslinking

**Figure 1.**
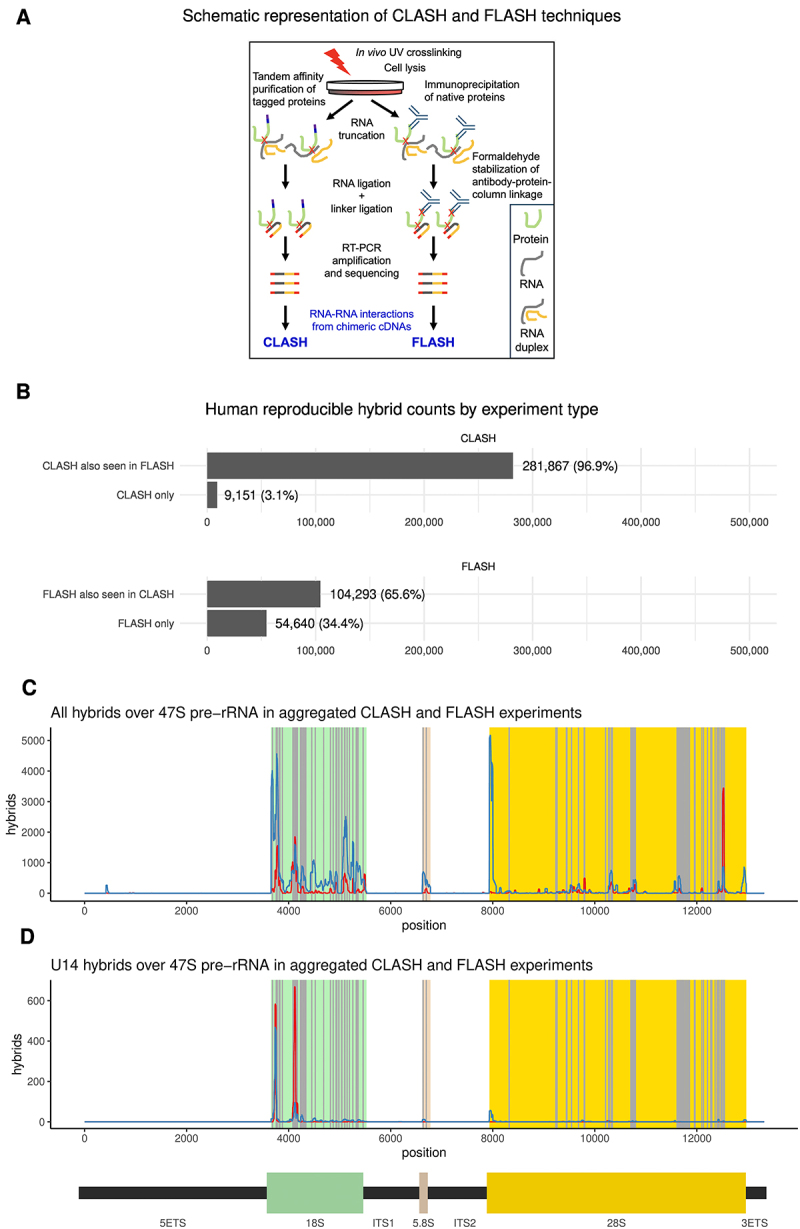
Overview of CLASH and FLASH results.

To identify potential novel snoRNA interactions, we initially applied the CLASH technique (Figure 1(A)) [38,42]. This involves UV crosslinking of RNA complexes with tagged proteins in living cells and tandem-affinity purification of the RNP complexes under stringent conditions. Ligation of linker adaptors is performed in parallel with internal ligation of captured RNA fragments base paired to each other. RNA is isolated, followed by reverse transcription and high throughput sequencing of cDNA libraries. Sequence data is then mapped to the genome. Most reads map to a single genomic site (termed ‘single reads’), representing the position at which the tagged protein was crosslinking to the transcriptome. However, if the crosslinked RNA forms part of a stable duplex, the ends of the two RNA species can be ligated together forming a chimeric cDNA. These are identified when two different regions of the resulting cDNA sequence map robustly to distinct sites in the genome (termed ‘hybrid reads’).

(A) Schematic representation of CLASH and FLASH techniques (see also Materials and Methods). For both approaches, live cells were UV-irradiated at 254nm to crosslink RNA–protein complexes. In CLASH, the cells used expressed FBL or NOP56 proteins carrying HTP tags (His6-TEV cleavage site-protein A), which were used for tandem affinity purification on IgG and Ni columns. Following TEV-mediated elution from the IgG column, RNA was partially digested and the RNA protein complexes were rebound to the Ni column in highly denaturing buffer containing 6M guanidinium HCl. On-column ligation was used to generate RNA-RNA chimeras and attach 5’ and 3’ linkers. RNA-protein complexes were eluted with imidazole. In FLASH, endogenous FBL protein was immunoprecipitated with a specific antibody, and complexes were bound to a protein A column. Following initial non-denaturing washing, interactions were stabilized by mild formaldehyde crosslinking, and the column was then washed with denaturing buffer containing 8M Urea. Following on-column ligation to generate RNA-RNA chimeras and attach 5’ and 3’ linkers, formaldehyde crosslinking was reversed and complexes eluted by heating to 100°C in buffer containing SDS. In both protocols, eluted RNA-protein complexes were gel purified, and proteins were then removed with proteinase K. Recovered RNAs were identified by sequencing, following reverse transcription and PCR amplification using the 5’ and 3’ linkers. Chimeric cDNAs represent the strands of RNA-RNA duplexes that had been ligated together, and were identified bioinformatically. (B) Reproducible hybrid counts by experiment type. Reproducibility of hybrids across CLASH and FLASH experiments in HEK293 cells. (C) All hybrids over 47S pre-rRNA in aggregated CLASH and FLASH experiments. The distributions of snoRNA hybrid hits in CLASH (red) and FLASH (blue) over 47S pre-rRNA show similar peaks, with known methylation sites shown in grey. (D) U14 hybrids over 47S pre-rRNA in aggregated CLASH and FLASH experiments. U14 (SNORD14) hybrids with rRNA in CLASH (red) and FLASH (blue) identify known interaction sites.

CLASH analyses require the use of a ‘bait’ protein fused to a tandem affinity purification tag. For these analyses, we tagged the core box C/D snoRNP proteins FBL or NOP56 with a C-terminal tag consisting of His6 – Precision protease cleavage site – Flag epitope. The tandem affinity tags allow very stringent purification of the bait protein, including highly denaturing conditions (6 M guanidinium HCl) to remove contaminating proteins (Figure 1(A)). The tagged proteins were previously shown to be functional in rRNA processing by rescue experiments, in which the endogenous proteins were depleted by RNAi [44]. Tagged constructs were integrated into the chromosome in Flip-in HEK293 cells at a pre-inserted LoxP site. The fusion proteins were expressed under a regulated pCMV-2xTET O_2_ promoter. Tetracycline levels were titrated to achieve expression close to the endogenous protein level, as assessed by western blotting (Fig. S1).

In order to assess robustness of our dataset we performed orthogonal validation, using formaldehyde assisted crosslinking ligation and sequencing of hybrids (FLASH) [36]. This approach is similar to CLASH in that RNA-protein interactions are captured by UV crosslinking in growing cells, but antibodies are used for immunopurification of endogenous RNA-protein complexes instead of tagged constructs. During purification, brief formaldehyde crosslinking is used to stabilize binding of the covalent bait protein-RNA complex to the protein A beads, allowing column washes under highly denaturing conditions (Figure 1(A)). Analyses of single hits for FBL and NOP56 showed that, for both proteins, snoRNA sites were most frequently recovered followed by rRNA and then mRNA hits, in both CLASH and FLASH analyses (Figure S1), supporting the reliability of the two complementary crosslinking approaches.

In human cells we recovered 591,958 hybrids overall (Table S1 and Dataset 1: https://data.mendeley.com/datasets/ptzhv3czxb/1). Recovered sequences that could be confidently mapped to two distinct regions of the genome (see Methods) were regarded as representing chimeric cDNAs resulting from RNA-RNA ligation. Non-identical chimeric sequences, or sequences recovered from different analyses, in which both segments overlapped were regarded as demonstrating independent recovery of the same interaction. The recovered RNA sequences were folded *in silico*, using the ViennaRNA Package 2.0 [45], to assess whether they arose from a stable RNA-RNA duplex. Interactions supported by at least two independent sequences, with a predicted ∆G of less than −12 Kcal mol^−1^, were considered stable and reproducible, and included in further analyses; this was the case for a total of 449,781 hybrids (Figure S3A). Among stable, reproducible hybrids, further filters were applied to ensure that hybrids called between snoRNAs and other classes of RNA were not mismapped internal snoRNA stems, and that hybrids between snoRNAs and RNAs that were not snoRNAs or rRNAs were not mismapped snoRNA-rRNA hybrids.

We compared CLASH from the cells expressing tagged Fibrillarin with FLASH from untagged control cells using anti-Fibrillarin antibodies. Strikingly, 97% of stable, reproducible RNA-RNA interactions recovered by CLASH were mapped to sites of interactions also recovered in FLASH (Figure 1(B)). More total hybrids were recovered with FLASH, and a lower fraction of those recovered by FLASH corresponded to interaction sites also found in CLASH data (66% of hybrids), with 34% FLASH only hybrids (Figure 1(B)).

The majority of hybrids mapping to snoRNAs were internal, representing stem structures (Figure S3; Table S1). These potentially allow visualization and analysis of snoRNA structures. Among intermolecular snoRNA hybrids, snoRNA-rRNA hybrids were most frequently recovered. From the set of stable, reproducible intermolecular hybrids after filtering, 69% were snoRNA-rRNA interactions, 9% were snoRNA-mRNA interactions, and 17% were snoRNA-snoRNA. It is notable that some highly abundant RNA species were recovered at low levels, in particular snoRNA-tRNA interactions represented only 0.7% of intermolecular hybrids, supporting the specificity of the interactions. The predominant recovery of snoRNA-rRNA interactions is consistent with the known function of snoRNAs in ribosome synthesis. Recovery of different RNA species in single reads (Figure S1) and chimeras (Figure S3) was in general agreement, supporting the recovery of authentic interactions.

To confirm the reliability of both methods we compared snoRNA-rRNA interactions recovered as hybrids in both types of experiments with the position of known rRNA methylation sites (Figure 1(C)). Comparing CLASH and FLASH results for snoRNA-rRNA targeting in human HEK cells we noticed that, although peak intensities varied to some extent, the same major interactions were recovered with both methods and correlated with known rRNA methylation sites. This was strongly supported by analyses of individual snoRNA interactions, e.g. U14, which is known to interact at two positions on the 18S rRNA sequence (Figure 1(D)). We conclude that both CLASH and FLASH provide consistent and reliable results. However, the background in FLASH analyses appeared higher than with CLASH, presumably reflecting the lower stringency of immunoprecipitation relative to tandem affinity purification.

### Identification of novel snoRNA-rRNA interaction sites

Modification sites in ribosomal RNAs have been well characterized by a variety of highly sensitive techniques, and it is very likely that all high-efficiency methylation sites have been identified (see [46,47] and references therein). However, rare modifications and other types of interactions can be envisaged, and we developed robust bioinformatics filters for snoRNA-rRNA interactions, to robustly categorize interaction classes.

**Figure 2.**
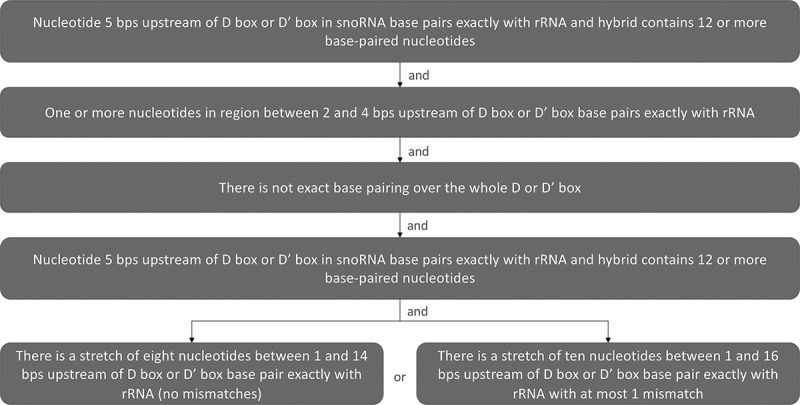
Decision tree used to categorize snoRNA-47S pre-rRNA hybrids.

We use the following strict filtering criteria to identify interactions that we can classify with high confidence as being capable of guiding methylation (Figure 2; see also Figure S11A below for filtering applied to U3):

The flowchart summarizes the decision rules.

The filtering criteria were applied to reproducible snoRNA-rRNA hybrids to identify those that are likely to guide methylation. See also Figure S21A below for filtering applied to U3.

- Nucleotide 5 bps upstream of D box or D’ box in snoRNA base pairs exactly with rRNA and hybrid contains 12 or more base-paired nucleotides
- One or more nucleotides in region between 2 and 4 bps upstream of D box or D’ box base pairs exactly with rRNA
- There is not exact base pairing over the whole D or D’ box
- Either:
  - There is a stretch of eight nucleotides between 1 and 14 bps upstream of D box or D’ box base pair exactly with rRNA (no mismatches), or
  - There is a stretch of eleven nucleotides between 1 and 16 bps upstream of D box or D’ box base pair exactly with rRNA with at most 1 mismatch

For each of the above criteria, G-U base pairs are not counted as exact base pairing.

**Figure 3.**
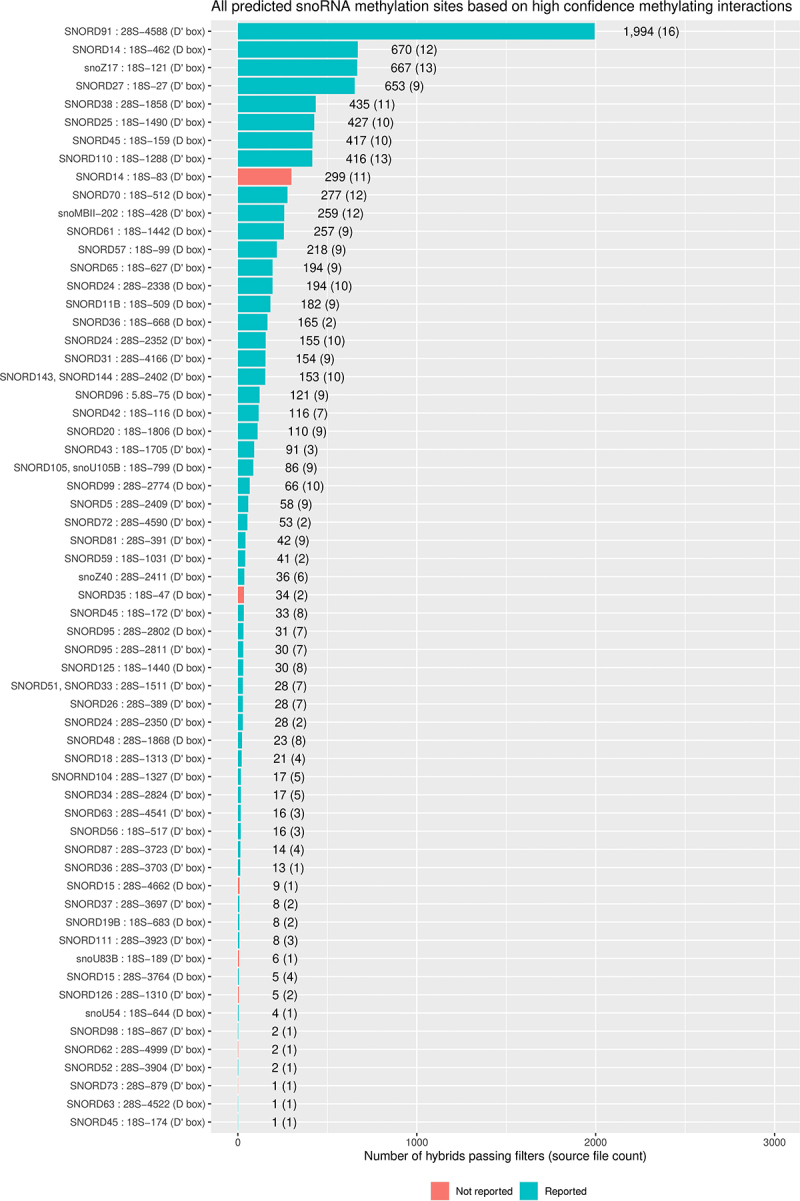
Distribution of predicted cognate snoRNA-rRNA interactions at methylation sites.

We recovered 9,363 hybrids passing these criteria and thus potentially able to guide methylation. In addition to known methylation sites we recovered novel snoRNA-rRNA interactions that could potentially guide methylation (Figure 3; Table S2). For SNORD14 (U14) interactions were recovered that would potentially guide methylation at 18S-462 and 18S-83. We conclude that the filters are quite conservative, and should recover interactions with a high likelihood of representing methylation-guide RNA binding sites.

Predicted sites of C/D box snoRNA guided methylation based on the filtered set of reproducible snoRNA-rRNA hybrids. The X axis labels show the snoRNA, the pre-47S transcript and co-ordinate at which methylation is predicted to occur, and the snoRNA box 5 base pairs downstream of the methylating nucleotide. The bars show the number of hybrids associated with the methylation event described in the access label, with the number of experiments in which relevant hybrids were found shown in brackets. Blue bars indicate previously reported methylation sites, and red bars indicate interactions that have not previously been reported to be associated with methylation. The Y axis lists snoRNA-rRNA interactions at sites of known or predicted sugar methylation.

Due to the strict criteria, many confirmed methylation site interactions were filtered out. We therefore also applied a weaker set of criteria to identify potentially methylating hybrids. These did not meet the strict base-pairing criteria, and allowed G-U base pairs, but the nucleotide 5 base pairs upstream of the D or D’ box did base pair with the rRNA. This ensured that hybrids that did not meet the strict criteria, but were associated with methylation site interactions, were not included in downstream analyses. This was done so that interactions that are expected to direct methylation, but do not fully match the bioinformatic criteria were not included as other types of interaction (e.g. blocking). We recovered 7,971 hybrids passing these weaker criteria, representing 412 interactions, including additional known methylation guide interactions for which no hybrids were found that passed the strict methylation criteria (Table S3).

Other hybrids did not meet the restricted or lenient methylation criteria, and whose pre-47S interacting fragment did not overlap with a nucleotide for which the snoRNA included in the hybrid was known to guide methylation. These were separated in three further categories: ‘ancillary’ (Table S4), ‘blocking’ (Table S5), or ‘structural’ hybrids (Table S6), depending on whether they potentially assist or interfere with the methylation function of a snoRNA, or might contribute to pre-rRNA folding during ribosomal subunit assembly (Figure 2).

Hybrids recovered 100 nt upstream or downstream of a methylation site directed by the same snoRNA, but not overlapping with it, were designated ‘ancillary’ as they could give additional structural support to the guide snoRNA interaction (Figure S4A; Table S1) [48]. We recovered 692 ancillary hybrids.

Hybrids formed by snoRNAs at methylation site, that are not predicted to guide methylation but form at least 22 perfect Watson-Crick pairs within 17 nt upstream or downstream of the site were called ‘blocking’ interactions as they might interfere with normal methylation. In total, 11,028 non-methylating hybrids overlapped with interactions guiding methylation (Table S1). Among these interactions, the majority guide methylation at neighbouring sites. It is unlikely that closely located sites (less than 20 nt separation) could be methylated simultaneously. Thus, over-expression of a snoRNA could lead to both increased methylation at its rRNA binding site and suppression at neighbouring sites. The presence of such overlapping methylation guide interaction sites suggests the need for a precise timing for snoRNA binding and methylation; such an ordered sequence may contribute to the correct folding of the pre-rRNAs and/or aid in avoiding kinetic traps [49,50]. For clarity, these interactions were not included in the ‘blocking’ interactions list. However, 62 high confidence blocking hybrids were identified for snoRNAs that are not predicted for direct methylation at closely located sites (Figure S4B; Table S1). Recent reports have highlighted the variability in methylation efficiency at different sites in the human rRNA [22,51–53], and we speculate that this may partly reflect competition for binding between snoRNA species. For instance, we observed interactions involving the abundant snoRNAs U3 and U8 that could block methylation sites. Our previous analyses in yeast also identified numerous, potentially regulatory interactions, particularly involving U3, suggesting that this is a conserved feature [37].

**Figure 4.**
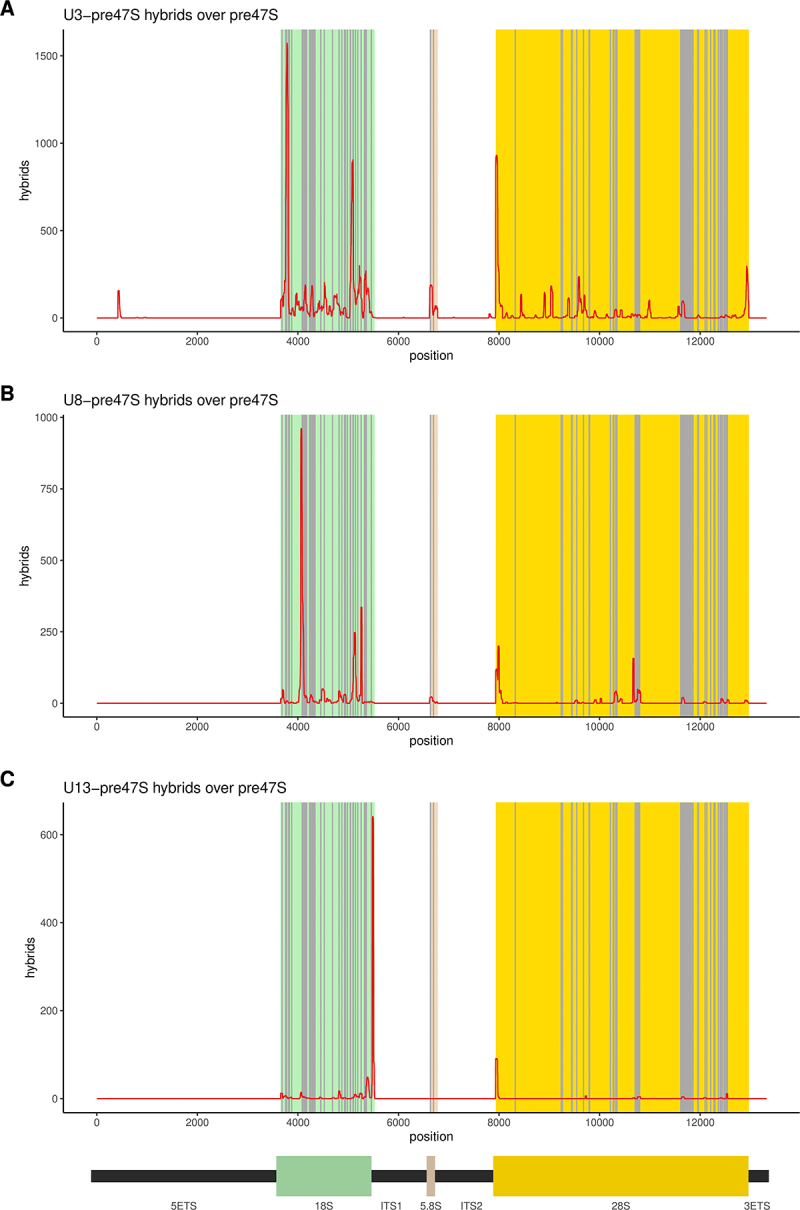
Distribution of interactions between select snoRnas and the 47S pre-rRNA.

All other confidently identified snoRNA-rRNA hybrids were termed ‘structural’ interactions, reflecting potential structural roles in supporting conformational changes and avoiding kinetic traps during pre-ribosome assembly and/or pre-rRNA folding [49,50]. These included a small number of box C/D snoRNAs implicated in ribosome synthesis steps other than rRNA methylation: U3, U8, U14 and the acetylation guide U13 (Figures 4(A-C) and S4C).

(A-C) The distribution of hybrid hits involving U3, U8, and U13, respectively, over the 47S pre-rRNA sequence. Reported methylation sites are shown in grey.

Among novel structural interactions, we found hybrids between 18S rRNA and the 3’ region of U8 snoRNA. It was previously reported that the 5’ end of U8 snoRNA is critical for 5.8S and 28S rRNA maturation [54]. However, no interactions for the 3’ region of U8 were described and the functional significance remains to be established. In *Xenopus*, the timing of association of the 3’ end of 5.8S rRNA and 5’ end of 28S was proposed to be regulated by initial binding of U8 at the 5’ end of 28S, promoting formation of a ‘bulge’ in the 28S sequence. This might act as a ‘priming site’ for base-pairing to 5.8S, leading to the eventual displacement of U8. We note that a peak of U8 interaction is located at the 5’ end of 28S (Figure 4(B)), potentially corresponding to this predicted interaction.

### Novel snoRNA-snoRNA interactions

Many reproducible snoRNA-snoRNA hybrids were recovered (261,166) (Table S1), but these predominately represented internal stems, particularly within U3 (229,525 hybrids). However, we also recovered 14,235 reproducible intermolecular hybrids between different snoRNA species, representing 1,422 distinct interactions (Figures S5A and S5B). These interactions suggest the existence of regulatory loops in snoRNA biogenesis and function, e.g. through possible titration/sequestration or ‘sponging’. In addition, a small number of interactions predicted to guide snoRNA methylation were detected (Figure S5C), using the same criteria as applied to rRNA (Figure 2).

### snoRNA-tRNA interactions

We noted that although tRNAs represented only a small proportion of all snoRNA hybrids (0.2%), they were enriched for interactions that potentially direct methylation. Overall, from 591 reproducible snoRNA-tRNA hybrids, two met the criteria for classification as high confidence methylating hybrids, and 161 met the criteria for classification as potentially methylating hybrids. Notably, for hybrids between snoRNAs and tRNAs that contain introns, 63% (71 out of 113 reproducible hybrids) were classified as high confidence or potentially directing methylation. In contrast, for hybrids between snoRNAs and tRNAs that do not contain introns, only 19% (92 of 478 reproducible hybrids) meet these criteria (Table S1).

Notably, SNORD97 and SCARNA97 (SNORD133) were reported to direct methylation of the wobble cytidine of human elongator tRNAMet(CAT) [55], and we found reproducible hybrids for both SNORD97 and SNORD133 with tRNA-Met-CAT-3–1 and tRNA-Met-CAT-1–1. Moreover, an snoRNA – tRNA interaction network was reported, that includes 32 snoRNAs [56]. We recovered snoRNA-tRNA hybrids for four of these snoRNAs, including SNORD97 and SNORD133, as well as SNORD33 and SNORD100, which interacted with tRNA-Gly-GCC-1–1. A link between SNORD33 and tRNA-Gly-GCC was also reported [56].

### snoRNA-mRNA interactions

Perhaps the most interesting class of snoRNA chimeras involved snoRNA-mRNA interactions. It has been proposed that snoRNAs can influence pre-mRNA splicing, processing and stability in mammalian cells; for examples see [13,25,39,57]. It is, however, also possible that snoRNAs might be ‘sponged’ on abundant mRNAs.

Among reproducible, stable hybrids, 28,120 snoRNA-mRNA hybrids were recovered, representing 1,755 interactions between 149 snoRNA families and 967 mRNAs. To eliminate potential mis-mapping errors we removed all hybrids that were called as snoRNA-mRNA hybrids, but whose mRNA fragment could also be aligned to U3 or to an rRNA sequence (albeit poorly). This filtering step retained 7,209 hybrids involving 117 snoRNAs and 940 mRNAs.

**Figure 5.**
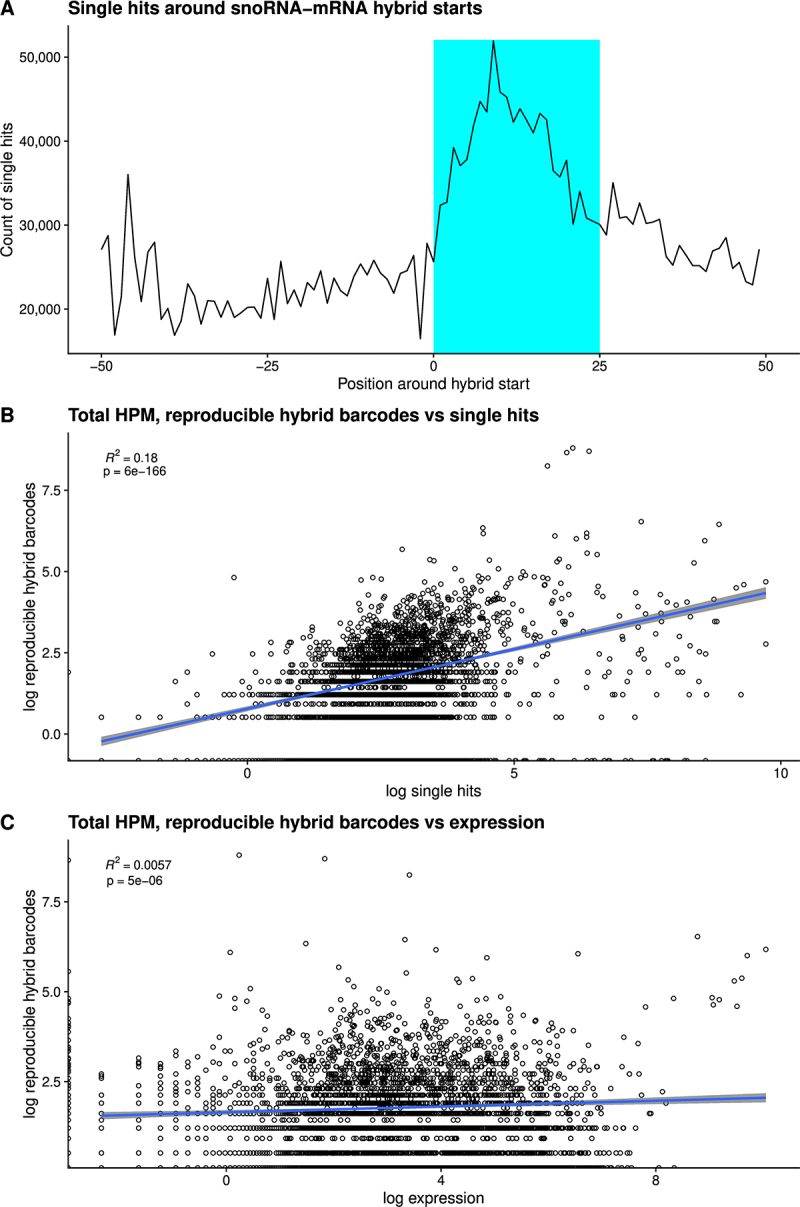
Single hits around snoRNA-mRNA hybrid starts, and correlation of snoRNA-mRNA hybrids with mRNA single hits and abundance.

The greatest number of filtered mRNA interactions was observed for U3 (50% of reproducible snoRNA-mRNA hybrids, interacting with 566 different mRNAs), followed by SNORD33 (8% of hybrids, 23 mRNAs), SNORD24 (3% of hybrids, 15 mRNAs), snoU83B (3% of hybrids, 63 mRNAs), and SNORD58 (3% of hybrids, 37 mRNAs). There was a clear enrichment for single hits around sites of snoRNA-mRNA hybrids (Figure 5A). The high correlation for mRNAs between filtered hybrids and single hits (*p* = 6e^−166^) confirmed enrichment of mRNA single hits in the regions of snoRNA interactions (Figure 5B). In contrast, there was little correlation between mRNA expression levels and recovery in snoRNA hybrids (R^2^ = 0.0057) (Figure 5C). These data support the conclusion that recovered mRNAs were specifically bound and represent *bona fide* interactions.

(A) Distribution of single hits around snoRNA-mRNA hybrid starts.

(B) Correlation between recovery in reproducible CLASH hybrids and mRNA single hits.

(C) Correlation between recovery in reproducible CLASH hybrids and mRNA abundance.

To assess whether novel snoRNA-mRNA interactions have the potential to direct RNA methylation, the hybrids were analysed using the same criteria as those applied to rRNA (Figure 2). This identified a small number of putative methylation guide interactions (Figure S8). In a GO term analysis on reproducible hybrids, the greatest enrichment was for cytoplasmic protein synthesis, followed by respiration (Fig. S9).

**Figure 6.**
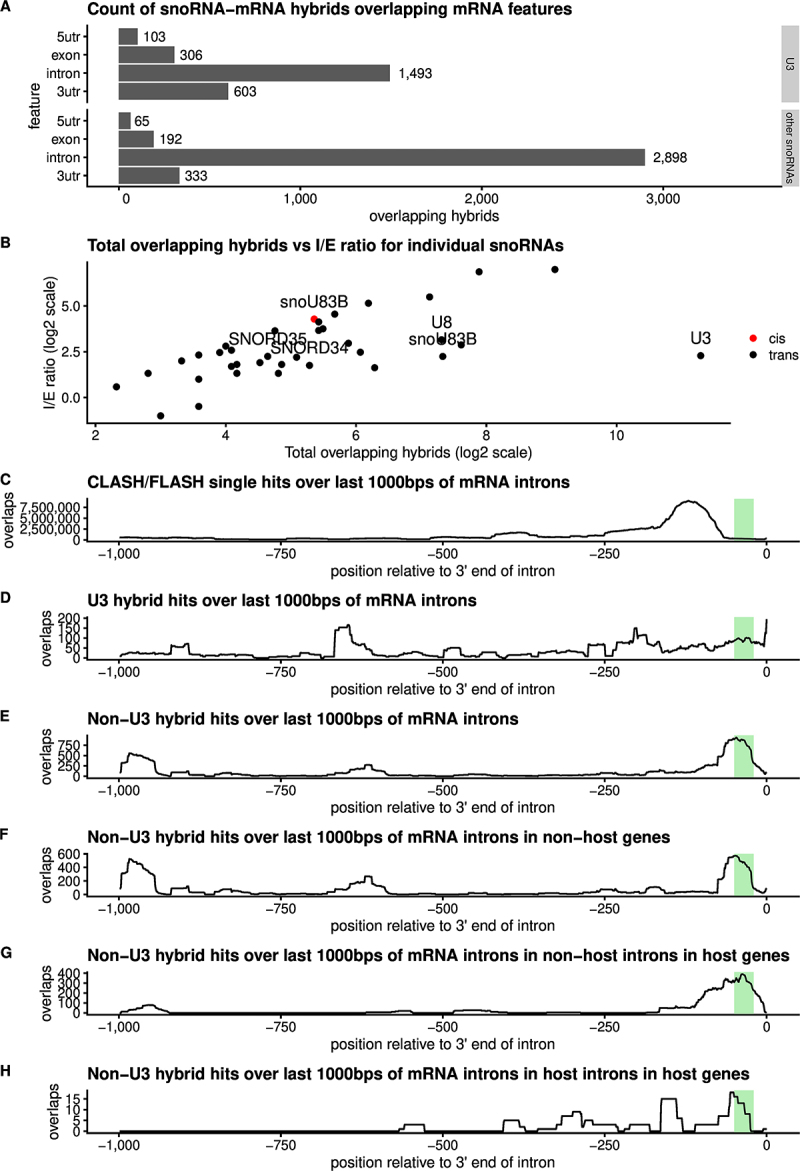
Distribution of snoRNA interactions on mRNAs.

Comparison of snoRNA-mRNA interactions revealed distinctly different patterns of interactions between U3 and other snoRNAs (Figure 6A and S10). Interactions with all snoRNAs were recovered in mRNA coding sequences (CDS) and untranslated regions (UTRs) but were substantially enriched in pre-mRNA introns (Figure 6A). However, more CDS interactions were recovered for U3 than for all other snoRNAs combined. Those U3 interactions that were identified within mRNA introns were predominately not in proximity to splice junctions. Moreover, most mRNA-U3 binding sites presented sharp peaks pointing to highly specific interactions. The strikingly high number of U3-mRNA interactions suggest a special role for U3 in mammalian gene expression, which might be reflected in the substantially greater abundance of U3 than other human snoRNAs and detection of stable abundant U3-derived fragments.

(A) Counts of hybrids overlapping different mRNA features for snoRNA−mRNA hybrids incorporating U3, and hybrids incorporating other snoRNAs, respectively. The plots show that while both classes of hybrid predominantly bind mRNA introns, hybrids incorporating U3 are significantly more likely to also bind other mRNA regions, in particular exons. (B) Total overlapping hybrids vs. intron / exon ratios for individual snoRNAs in *cis-* and *trans-*. (C-H) CLASH / FLASH hits over the last 1000 base pairs of mRNA introns, with the estimated branch point region (20-50 bps upstream of the 3’ end of the intron) highlighted in green. Non-U3 hybrids show a pronounced peak overlapping the estimated branch point region in host introns, non-host introns within the host gene, and introns within non-host genes.

To study the functional role of U3 interactions, we depleted U3 from HEK cells with morpholino anti-sense oligos (ASOs). We previously reported that U3 depletion using these ASOs impaired pre-rRNA processing by 48 h [58]. In the current study we performed Bulk RNA-Seq and performed differential expression analysis, comparing cells that were U3-depleted for 2 days and cells that were U3-depleted for 3 days with control (mocktreated) cells. The results of our differential expression analyses are shown in figure S10. As the figure shows, U3-depletion resulted in significant changes in expression for a large number of genes in both directions. In all, 23% of mRNAs were significantly differentially expressed after 2 days, and 29% were significantly differentially expressed after 3 days. In each case, a significantly higher proportion of mRNAs identified as U3 interactors in CLASH showed altered levels than total mRNAs (Chi Square Test; *p* = 4e^−6^ after 2 days, *p* = 3e^−7^ after 3 days). U3 bound mRNAs showed both increased and decreased levels, but a particularly high proportion of CLASH/FLASH U3 interactors showed reduced abundance after both 2 days and 3 days of U3 depletion (20% of U3 interactors compared to 12% of mRNAs overall after 2 days, Chi Square Test; *p* = 7e^−6^, and 25% of U3 interactors compared to 15% of mRNAs overall after 3 days, Chi Square Test; *p* = 2e^−7^). We note that changes in mRNA abundance are likely a mixture of direct effects and indirect consequences of impaired ribosome synthesis. We performed GO enrichment analysis of differentially expressed U3 interactors and differentially expressed mRNAs after 2 and 3 days. The results of these analyses for 3 days of depletion are shown in Figure S13. Overall, mRNAs showing elevated expression following U3 depletion were enriched for ribosome synthesis factors. Whereas the most depleted GO term was macroautophagy. Among U3 interacting mRNAs, cytoplasmic translation-related function was both the most upregulated and down regulated GO term.

Notably, we observed a correlation between the presence of a snoRNA interacting with U3 in the intron and levels of its host mRNA after U3 depletion, pointing to general interplay between biogenesis of snoRNAs and their host genes. Initial comparison of the binding sites to the patterns of evolutionary conservation across 23 mammalian species did not identify enrichment for conserved regions in snoRNA binding sites relative to their flanking regions. However, further analysis revealed that the fall in conservation was due to the frequent presence of an exon near the snoRNA binding site. The 3’ region of introns that bind snoRNAs was more conserved than, for instance, the 5’ region of the same intron not harbouring interactions. This observation supports the functional importance of the interactions (Figure S7).

### Binding is frequently found within introns that host snoRNAs

We noted that a substantial proportion of snoRNA hybrids with intronic regions represented interactions between snoRNAs and their host introns (5% or 220 hybrids), suggesting frequent connections between snoRNA biogenesis and host gene splicing (Figure 6). Around 16% of all C/D snoRNA-mRNA interactions were represented by peaks towards the 3’ ends of introns, *in cis* or *in trans*, in the region of potential intron branch point (25 to 50 bp from the 3’ SS) (Figure 6) pointing to the possibility of their involvement in splicing of the bound mRNAs.

**Figure 7.**
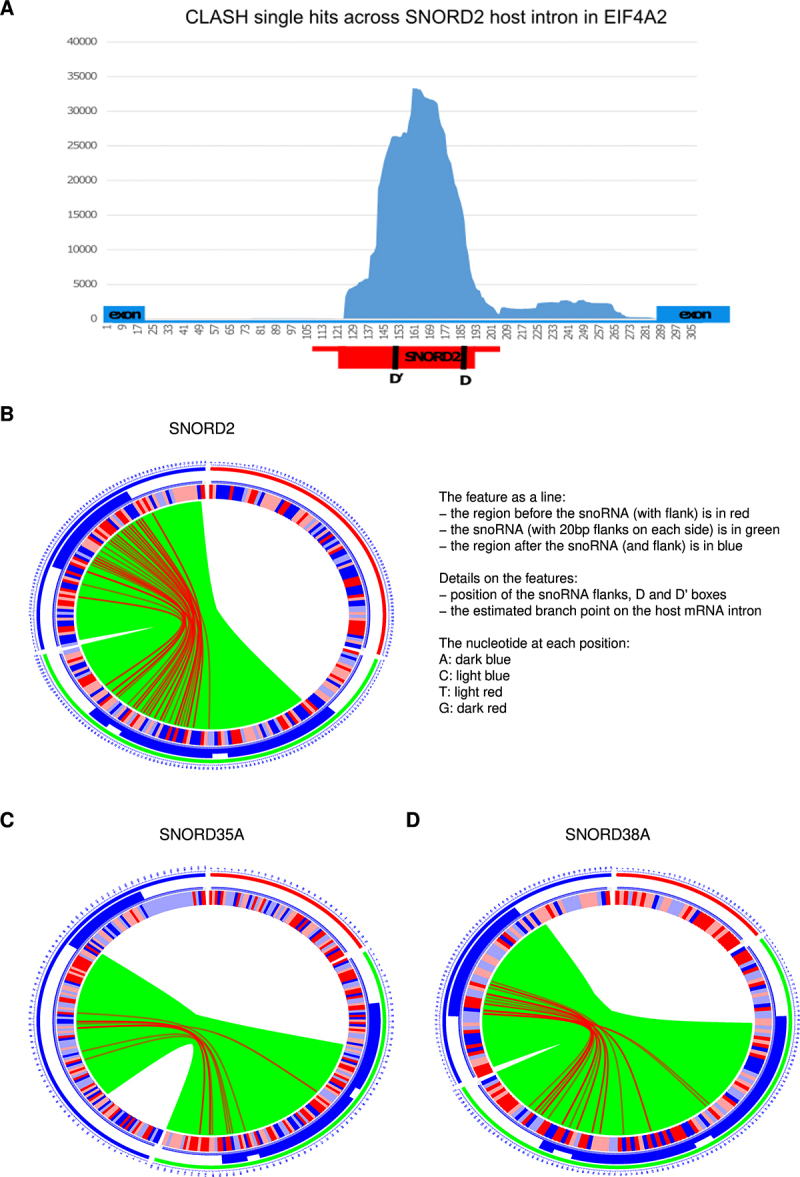
Interactions between selected snoRNAs and host introns.

Intronic snoRNAs frequently formed predicted duplexes with the 3’ flanking region that included the intron branch point and/or of the polypyrimidine tract, both of which are important signals for pre-mRNA splicing. For examples see Figure 7 and S10B. We speculate that these interactions may slow pre-mRNA splicing allowing sufficient time for assembly of snoRNP proteins with the nascent transcript prior to pre-mRNA intron excision and digestion to the mature snoRNA ends.

(A) Distribution of CLASH single hits across the host intron of SNORD2 in EIF4A2, showing that reads that do not overlap the snoRNA overlap the 3’ end of its host intron (B,C) Circos plots showing hybrids between SNORD2, SNOR35A, and SNORD38A, and their host introns. In each case, hybrids are exclusively formed between the snoRNA and the 3’ end of its host intron.

## Discussion

Here we systematically mapped the interactions between box C/D snoRNAs and the human transcriptome, using UV crosslinking followed by generation and sequencing of RNA hybrids. To achieve high specificity and robustness, we used two complementary approaches: CLASH relies on the expression of fusion proteins including high-affinity purification tags, suitable for extensive washes in denaturing conditions, whereas FLASH utilizes specific antibodies against the native proteins, in combination with mild chemical crosslinking with formaldehyde to stabilize the interactions. Comparison of snoRNA hybrids recovered with CLASH and FLASH revealed a high degree of overlap (Figure 1B), and we therefore combined these datasets for most analyses.

We previously established that CLASH performed in HEK cells faithfully recovers *in vivo* interactions [42] and this conclusion is strongly supported by the predominance of interactions recovered with pre-mRNA introns relative to mRNA exons. In total RNA, exons are much more abundant than introns, indicating that *in vitro* contacts formed after lysis are not a major source of the hybrids recovered. We also note that very few snoRNA-tRNA interactions were recovered, despite the high abundance of tRNAs in total RNA.

As expected, we recovered many interactions between the cognate snoRNAs and known or predicted sites of rRNA methylation. Recovery of different snoRNAs was quite variable and we speculate that this may partially reflect the residence time of the snoRNA species on the pre-rRNA. Moreover, it was notable that at many sites of methylation of the human rRNA, multiple snoRNAs could be identified with complementary base-pairing that matched the features previously defined as required for 2’-*O*-methylation: ≥12 bp showing perfect complementarity, with the modified nucleotide positioned 5 bp from a site D motif [59]. These data indicate a high degree of redundancy in human snoRNA-rRNA interactions. Quantitative analyses of human rRNA methylation identified many sites that show partial methylation, suggesting that functionally distinct ribosomes might be generated under different growth conditions [51–53,60–62]. *In vivo* differences were largely linked to growth rate and tumour types [61], suggesting that these features may underpin heterogeneity observed in cell lines.

We also found numerous cases in which snoRNAs were recovered bound to the pre-rRNA close to methylation sites with strong Watson-Crick base-pairing, but in a configuration that is not expected to guide RNA modification. We speculate that these interactions regulate the timing and/or efficiency of rRNA modification by competing with cognate methylation-guide snoRNAs. In addition, they may contribute to pre-rRNA folding dynamics during ribosomal subunit biogenesis, as previously proposed [49,50]. The emergence of snoRNAs with overlapping specificities and overlapping binding sites, indicate that the site-specific regulation of rRNA methylation is both functionally important and complex.

A recent analysis applied chimeric eCLIP to identify snoRNA interactions in mouse and human cells, including HEK293 [40]. 82% (77 out of 94) of the snoRNA-rRNA interactions and 66% (4 out of 6) snoRNA-snRNA interactions reported in HEK cells by Song et al. were also present in our dataset.

Many reproducible snoRNA hybrids represented interactions with mRNAs or pre-mRNAs. There was modest overlap between mRNA sites identified as methylated by Nm seq [63,64] and snoRNA interaction sites: 23 in HEK cells, and 19 in Hela cells. Most relevant interactions were recovered multiple times (Table S7), however, the predicted base pairing did not clearly match the consensus for methylation site selection.

Human U3 is around 5- to 50-fold more abundant than most methylation-guide snoRNAs and was responsible for 50% of all filtered snoRNA-mRNA hybrids. Hybrids with pre-mRNAs were predominately involved a 3’ region of U3, which is distinct from the 5’ sequences involved in the interactions with pre-rRNAs. The major region of U3 recovered in mRNA interactions corresponds to the methylation-guide region adjacent to box D in other snoRNAs. Notably, the homologous 3’ region of U3 in budding yeast also forms numerous mRNA interactions [37,38]. This suggests an evolutionarily conserved alternate fold for yeast and human U3, distinct from the ‘hinge-mediated’ interactions with the pre-rRNA.

Following U3 depletion, mRNAs identified as bound by U3 were highly over-represented among mRNAs showing altered abundance (Figure S12). However, the mechanistic links between U3 interactions and altered mRNA abundance remain unclear. Fragmentation of snoRNAs into miRNA-like species has been reported [65,66]. However, we did not observe accumulation of short (≤22nt), miRNA-like fragments of snoRNAs in single reads, and the average length distribution of the snoRNA fragment of snoRNA-mRNA chimeras was around 35 to 40-nt. We conclude that snoRNA-mRNA interactions do not resemble those formed by miRNAs in structure, distribution or, most likely, in function.

The snoRNAs are normally nuclear restricted, and show strong nucleolar enrichment but are in rapid exchange with nucleoplasmic pools [67]. Consistent with interactions formed within the nucleus, interaction sites for U3 and other snoRNAs were strongly enriched for pre-mRNA introns (Figure 6A). Pre-mRNA splicing generally occurs cotranscriptionally or rapidly follows transcript release. This strongly indicates that most interactions are formed between snoRNAs and nuclear pre-mRNAs at or close to the sites of transcription. It could be envisaged that snoRNA binding might promote stabilization or destabilization of interacting pre-mRNAs, or alter processing and/or transport efficiency, depending on the precise interaction sites.

There have been reports of snoRNA detection in the cytoplasm (reviewed in [16]) and we cannot exclude the possibility that some exon interactions occur with mature cytoplasmic mRNAs. Following nuclear breakdown during mitosis, U3 and other nucleolar components relocalize to the surface of the condensed mitotic chromosomes (reviewed in [68,69]), raising the further possibility of localized mRNA interactions. The intrinsically disordered regions of the snoRNP proteins are reported to form condensates under conditions of nutritional stress [70]. However, growth conditions used here are not nutrient limited, so stress-induced condensation seems unlikely to be a major driver of the interactions we observe.

In human cells, the majority of snoRNA genes are located within introns in protein coding genes. It was previously suggested that processing of snoRNAs and splicing of the host gene may be connected [11,71,72]. Indeed, folding of the snoRNA snoRD86, which is encoded in an intron of the NOP56 gene, acts as sensor in controlling the abundance of this snoRNP core protein [73]. We observed that intronic regions flanking snoRNAs were frequently recovered in hybrids with the same snoRNA, as well as with other snoRNAs. We suggest that such interactions facilitate coordination between the maturation of snoRNA and splicing of the host gene. Debranched introns are expected to be rapidly degraded by both the 5’ exonuclease Xrn2 and 3’ exonucleases of the exosome complex. It is therefore important that snoRNA folding and snoRNP assembly precedes intron excision. The observed interactions may coordinate these steps. A similar conclusion was independently reported [11].

Despite the essential roles they play during ribosome biogenesis through their involvement in pre-rRNA modification, processing, and folding, it remains unclear to what extent box C/D snoRNAs contribute to regulating the homeostasis of other cellular RNAs, including mRNAs. This is particularly relevant for abundant species such as U3. The data on interaction sites reported here may aid the elucidation of non-conventional roles of box C/D snoRNAs and the potential links between altered expression and differentiation, development or disease.

## Materials and methods

### Human cell culture and UV crosslinking

Human embryonic kidney HEK cells were grown to 80% confluency in DMEM, 10%FBS medium and were UV crosslinked on ice with λ = 254 nm in Stratalinker 1800 (Stratagene), at 400 mJ/cm2. U3 was depleted from HEK cells by use of a chimeric antisense oligonucleotide, as described [58].

### Hek cell lysis

HEK cells were lysed by addition of ice-cold TM150 buffer (20 mM Tris-HCl pH 7.4, 150 mM NaCl, 0.4% NP-40, 2 mM MgCl2, 1 mM DTT, protease inhibitors (Roche, complete, EDTA-free), RNase Inhibitor (Promega)). 10 u of RQ1DNAse (Promega) were added, the samples were mixed by pipetting and incubated for 10 min at room temperature to break genomic DNA and to ease the extraction of nuclear Fibrillarin binding complexes. Lysates were centrifuged in Eppendorf mini centrifuge at 14000rpm and 4C for 10 min and supernatant was collected.

### First affinity purification

In human HEK CLASH, beads conjugated with anti-Flag M2 (Sigma) were used: in FLASH, Protein A agarose conjugated with IgG and anti-FBL AB was used. Cell lysates were incubated with beads for 60 min at 4°C. Supernatant was discarded and the recovered beads were washed twice with PBS-WB buffer (PBS, plus 150 mM NaCl, 2 mM MgCl2, 0.4% NP-40), and once in 1X PBS, 2 mM MgCl2.

### RNase treatment and formaldehyde crosslinking

RNP complexes bound to the beads were treated with 0.5 units RNaseA+T1 mix (RNace-IT, Stratagene) in 100 μl PBS,2 mM MgCl2 buffer for 10 min at 20°C. In CLASH 900 μl GDB denaturing buffer (6 M GuCl2, 150 mM NaCl, 20 mM Tris pH = 7.4) was added to the beads with RNase and mixed thoroughly. Supernatant with denatured complexes was removed and added to Ni beads (Gibco) washed in GDB, with subsequent binding carried out for 1 h at 4C. In FLASH, to remove indirect RNA and protein binding from the complexes the beads were washed twice with PBS-WB buffer (PBS, +150 mM NaCl, 2 mM MgCl2, 0.4% NP-40), twice with HS-PBS-WB (PBS, 0.3 M NaCl, 2 mM MgCl2, 0.4% NP-40) and once in 1xPBS. The complexes were cross linked on beads in 0.2% formaldehyde in PBS for 3 min, then formaldehyde was quenched by addition of glycine to 0.2 M and Tris-HCl pH = 8 to 0.1 M and incubation for 5 min. Crosslinked complexes were subjected to 4x denaturing washes in UB (20 mM Tris pH = 7.4, 8 M UREA, 0.3 M NaCl, 0.4% NP-40) and additional incubation in UB for 30 min at 4C to remove non-specific interactions. Subsequent steps were identical for FLASH and CLASH. We performed 15 CLASH experiments and 3 FLASH experiments. Details can be found in the GEO record: https://www.ncbi.nlm.nih.gov/geo/query/acc.cgi?acc=GSE114825

### RNA end modification

In CLASH, Ni beads were washed twice with GDB buffer, twice with TM150, twice with PNK buffer (50 mM Tris-HCl pH 7.5, 10 mM MgCl_2_, 0.5% NP-40, 50 mM NaCl).

In FLASH, beads with bound RNA-protein complexes were washed 4x with PNK buffer.

To remove unwanted 3’phosphate groups from bound RNA fragments in both CLASH and FLASH the complexes were treated with TSAP phosphatase (Promega) using provided buffer for 40 min at room temperature. To inactivate the enzyme the beads were washed twice with UB (FLASH) or GDB (CLASH) and 4x with PNK buffer.

Then the 5’ phosphorylation and radioactive labelling of RNA were carried out. The complexes on the beads were incubated with 40 units T4 Polynucleotide kinase (New England Biolabs), first with P32 labelled ATP for 45 min, then 20 more min with 1 mM cold ATP, in PNK buffer with RNase inhibitors (RNasin, Promega) at room temperature. The reaction should provide 5’ phosphates needed for downstream ligations. The beads then were washed as before, twice with UB (FLASH) or GDB (CLASH) and 4x PNK buffer.

### Linker ligation and RNA-protein complex elution

Protein-bound RNA molecules were ligated together and with 3’ linker (1 μM miRCat-33, IDT), overnight using 40 units of T4 RNA ligase 1 (New England Biolabs) in PNK buffer with RNase inhibitors at 16°C. This reaction created RNA hybrids and single RNA molecules ligated to miRCat linker. On the next day, the beads were washed as before 2x UB (FLASH) or GDB (CLASH) and 4x PNK buffer. Then using 40 units of RNA ligase 1, barcoded 5′ linkers (final conc. 5 μM; IDT, one for each sample) were ligated in RNA ligase 1 buffer with 1 mM ATP for 3–6 h at 20°C. The beads were washed as before. In CLASH the complexes were eluted in EB (2x NuPage Sample buffer, 400 mM Imidazole, 10 mM Tris-HCl pH = 7.4, 10 mM DTT). In FLASH the complexes were washed off the beads by partial destruction of formaldehyde crosslinking by boiling the samples in NuPAGE protein sample buffer plus 100 mM Tris-HCl, 1%SDS, 100 mM ME (β-mercaptoethanol) for 3 min. The supernatant with RNA-protein complexes was recovered from cooled samples.

### SDS-PAGE, and transfer to nitrocellulose

Protein-RNA complexes in NuPAGE SB plus SDS, ME (Life Technologies) were resolved on a 4%–12% Bis-Tris NuPAGE gel (Life Technologies) in NuPAGE SDS MOPS running buffer then they were transferred to nitrocellulose membrane (GE Healthcare, Amersham Hybond ECL) in NuPage transfer buffer (Life Technologies) with 10% methanol for 1 hr at 100 V. Depending on the strength of the signal the membrane was exposed on film (Amersham) for 1 hr or overnight at −70C. Developed film was aligned with the membrane and the radioactive bands corresponding to the Fibrillarin-RNA complexes were excised.

### Proteinase K treatment, RNA isolation and cDNA library preparation

Cut out bands were incubated with 150 μg of Proteinase K (Roche) and proteinase K buffer (50 mM Tris-HCl pH 7.8, 50 mM NaCl, 0.4% NP-40, 0.5% SDS, 5 mM EDTA) for 2 hr at 55°C. The RNA was extracted with phenol-chloroform-isoamyl alcohol (PCI) mixture and ethanol precipitated overnight with 10 μg Glycogen (Ambion, Life Technologies). The isolated RNA was dissolved in 12 mkL of distilled RNAse-free water and reverse transcribed using miRCat-33 primer (IDT) with Superscript III Reverse Transcriptase (Life Technologies) in its buffer for 1 h at 50°C. RNA was then degraded by addition of RNase H (New England Biolabs) for 30 min at 37°C. cDNA was amplified using primers P5 and primer PE_miRCat_PCR and TaKaRa LA Taq polymerase (Takara Bio). PCR products were separated on a 2% MetaPhor agarose (Lonza) gel with SYBRSafe (Life Technologies) in 1 ×TBE at +4C. The gel band corresponding to 150-200bp was cut out. cDNA was purified with MinElute Gel Extraction Kit (QIAGEN). Obtained cDNA libraries were sent for high-throughput sequencing.

### Oligonucleotides

#### 3’ linker

miRCat-33 linker (IDT) AppTGGAATTCTCGGGTGCCAAG/ddC/

#### 5’ linkers

L5Aa invddT-ACACrGrArCrGrCrUrCrUrUrCrCrGrArUrCrUrNrNrNrUrArArGrC-OH L5Ab invddT-ACACrGrArCrGrCrUrCrUrUrCrCrGrArUrCrUrNrNrNrArUrUrArGrC-OH

L5Ac invddT-ACACrGrArCrGrCrUrCrUrUrCrCrGrArUrCrUrNrNrNrGrCrGrCrArGrC-OH

L5Bb invddT-ACACrGrArCrGrCrUrCrUrUrCrCrGrArUrCrUrNrNrNrGrUrGrArGrC-OH

L5Bc invddT-ACACrGrArCrGrCrUrCrUrUrCrCrGrArUrCrUrNrNrNrCrArCrUrArGrC-OH

L5Bd invddT-ACACrGrArCrGrCrUrCrUrUrCrCrGrArUrCrUrNrNrNrUrCrUrCrUrArGrC-OH

L5Ca invddT-ACACrGrArCrGrCrUrCrUrUrCrCrGrArUrCrUrNrNrNrCrUrArGrC-OH

L5Cb invddT-ACACrGrArCrGrCrUrCrUrUrCrCrGrArUrCrUrNrNrNrGrGrArGrC-OH

L5Cc invddT-ACACrGrArCrGrCrUrCrUrUrCrCrGrArUrCrUrNrNrNrArCrTrCrArGrC-OH

L5Cd invddT-ACACrGrArCrGrCrUrCrUrUrCrCrGrArUrCrUrNrNrNrGrArCrTrTrArGrC-OH

The fixed barcodes are underlined. N indicates mixed nucleotides for random barcodes.

### PCR primers

miRCat-33 primer (IDT) CCTTGGCACCCGAGAATT primer for RT

PE_miRCat_PCR CAAGCAGAAGACGGCATACGAGATCGGTCTCGGCATTCCTGGCCTTGGCACCCGAGAATTCC library amplification

P5 AATGATACGGCGACCACCGAGATCTACACTCTTTCCCTACACGACGCTCTTCCGATCT library amplification

### Bioinformatics

#### Analysis of clash data

Raw sequences were preprocessed prior to alignment using hyb [74] by running the hyb preprocess command with standard parameters. The preprocessed data were aligned to a custom database combining multi-exon transcripts and unspliced genes (with snoRNA genes extended by 20bps in each direction and masked out of the genes in which they are contained where appropriate). The custom database was built using reference data from Ensembl release 77 (www.ensembl.org). To facilitate the analysis of snoRNA/rRNA hybrids, the complete human ribosomal DNA repeating unit (https://www.ncbi.nlm.nih.gov/nuccore/U13369) was also included in the database. Sequence alignment was performed using the blastall command, using the standard parameters from the hyb pipeline [74]. The aligned reads were processed using a variant of the hyb pipeline, modified slightly to extract snoRNA hybrids rather than microRNA hybrids preferentially. Hybrids identified using this process were then filtered to exclude sequences that could be aligned as single reads to the human genome (Ensembl release 77) using Novoalign 2.07 (www.novocraft.com) to prevent single reads overlapping gene boundaries from being mistakenly identified as hybrids. Downstream analysis was performed on reproducible hybrids (in which both fragments were found to overlap in two or more hybrids) with a predicted folding energy of −12dG or below. Among these stable, reproducible hybrids, further filters were applied to ensure that hybrids between snoRNAs and other classes of RNA were not mismapped U3 stems, and that hybrids between snoRNAs and RNAs that were not snoRNAs or rRNAs were not mismapped snoRNA-rRNA hybrids. The analysis was performed using the hybtools python package (http://www.github.com/hyweldd/hybtools), which was developed for this project. Reference data for the analysis of human rRNA methylation sites were obtained from [52] and [32].

The mRNA interaction sites were compared to reported human mRNA methylation sites [63,64] (Table S7). Genomic co-ordinates were identified for the mRNA fragments of all reproducible snoRNA-mRNA hybrids after ribosomal filtering and U3 filtering. For each of these fragments, numbers of (unstranded) genomic overlaps with methylation sites found using Nm-Seq in HEK cells or HeLa cells were determined using Bedtools intersect. 3. Files were filtered to include only mRNA fragments of hybrids identified as high confidence methylating hybrids or potentially methylating hybrids. For each of these fragments, closest methylation site reported from Nm-Seq in HEK cells using Bedtools closest. The GO term analysis was performed using clusterProfiler [75].

All interactions recovered are listed in Dataset 1.

### Analysis of RNA-Seq data

RNA-Seq data were processed using STAR [76]) and DESeq2 [77], using a human genome database from Ensembl release 77 (www.ensembl.org).

## Supplementary Material

Dunn Davis et al. Supp. Tables S1-S7.docx

Dunn Davis et al. Supp. Figs S1 - S13.docx

## Data Availability

All sequence data from this study have been submitted to the NCBI Gene Expression Omnibus (GEO). www.ncbi.nlm.nih.gov/geo/query/acc.cgi?acc=GSE114825 www.ncbi.nlm.nih.gov/geo/query/acc.cgi?acc=GSE121414 www.ncbi.nlm.nih.gov/geo/query/acc.cgi?acc=GSE121415 Dataset 1, listing all recovered interactions, is available from Mendeley data: https://data.mendeley.com/datasets/ptzhv3czxb/1 All other data are freely available on reasonable request to the authors. A pre-print version of this paper was previously made available [78].
