# Supplementary material for: Systematic mapping of small nucleolar RNA interactions in human cells
Source: RNA Biol. 2025 Nov 14;22(1):1–22. doi: 10.1080/15476286.2025.2589573 (PMC12645875; doi:10.1080/15476286.2025.2589573)
Supplement: Dunn Davis et al. Supp. Tables S1-S7.docx [file KRNB_A_2589573_SM0068.docx]

Systematic mapping of small nucleolar RNA targets in human cells

Supplementary tables:

Table S1: Overall hybrid counts by biotype 2

Table S2: High confidence methylating hybrids 5

Table S3: Potentially methylating hybrids 7

Table S4: Ancillary hybrid counts 15

Table S5: Blocking hybrid counts 18

Table S6: Structural hybrid counts 19

Table S7: snoRNA interactions overlapping mRNA methylation sites 20

# Table S1: Overall hybrid counts by biotype

| Interaction | Total unfiltered hybrids | Filtered hybrids | Filters applied |
| --- | --- | --- | --- |
| snoRNA:::snoRNA | 281,806 | 261,166 | Reproducibility, Stability |
| lincRNA:::lincRNA | 79,069 | 67,342 | Reproducibility, Stability |
| snoRNA:::rRNA | 73,374 | 57,069 | Reproducibility, Stability, U3 |
| mRNA:::mRNA | 58,557 | 17,354 | Reproducibility, Stability |
| snoRNA:::mRNA | 52,819 | 7,209 | Reproducibility, Stability, U3, rRNA |
| lincRNA:::mRNA | 17,190 | 4,191 | Reproducibility, Stability |
| lincRNA:::Mt-rRNA | 4,461 | 3,012 | Reproducibility, Stability |
| snoRNA:::lincRNA | 4,277 | 676 | Reproducibility, Stability, U3, rRNA |
| snoRNA:::Mt-rRNA | 3,030 | 1,692 | Reproducibility, Stability, U3, rRNA |
| Mt-rRNA:::Mt-rRNA | 2,177 | 1,079 | Reproducibility, Stability |
| misc-RNA:::misc-RNA | 1,274 | 1,257 | Reproducibility, Stability |
| mRNA:::Mt-rRNA | 1,208 | 141 | Reproducibility, Stability |
| snoRNA:::tRNA | 947 | 591 | Reproducibility, Stability, U3, rRNA |
| antisense:::antisense | 925 | 672 | Reproducibility, Stability |
| snoRNA:::snRNA | 901 | 306 | Reproducibility, Stability, U3, rRNA |
| lincRNA:::tRNA | 860 | 454 | Reproducibility, Stability |
| snoRNA:::antisense | 739 | 100 | Reproducibility, Stability, U3, rRNA |
| lincRNA:::Mt-tRNA | 717 | 227 | Reproducibility, Stability |
| snoRNA:::Mt-tRNA | 677 | 385 | Reproducibility, Stability, U3, rRNA |
| lincRNA:::snRNA | 672 | 315 | Reproducibility, Stability |
| snoRNA:::misc-RNA | 643 | 418 | Reproducibility, Stability, U3, rRNA |
| lincRNA:::misc-RNA | 622 | 394 | Reproducibility, Stability |
| antisense:::lincRNA | 542 | 164 | Reproducibility, Stability |
| processed-transcript::: processed-transcript | 509 | 159 | Reproducibility, Stability |
| antisense:::mRNA | 471 | 45 | Reproducibility, Stability |
| lincRNA:::processed-transcript | 383 | 109 | Reproducibility, Stability |
| snoRNA:::processed-transcript | 339 | 65 | Reproducibility, Stability, U3, rRNA |
| mRNA:::processed-transcript | 309 | 33 | Reproducibility, Stability |
| lincRNA:::rRNA | 263 | 129 | Reproducibility, Stability |
| mRNA:::tRNA | 255 | 60 | Reproducibility, Stability |
| mRNA:::Mt-tRNA | 217 | 23 | Reproducibility, Stability |
| misc-RNA:::mRNA | 215 | 31 | Reproducibility, Stability |
| mRNA:::snRNA | 163 | 29 | Reproducibility, Stability |
| Mt-rRNA:::Mt-tRNA | 104 | 20 | Reproducibility, Stability |
| lincRNA:::sense-overlapping | 102 | 25 | Reproducibility, Stability |
| lincRNA:::miRNA | 90 | 21 | Reproducibility, Stability |
| snoRNA:::sense-overlapping | 85 | 20 | Reproducibility, Stability, U3, rRNA |
| miRNA:::mRNA | 79 | 13 | Reproducibility, Stability |
| miRNA:::miRNA | 59 | 52 | Reproducibility, Stability |
| tRNA:::tRNA | 57 | 32 | Reproducibility, Stability |
| mRNA:::sense-overlapping | 55 | 3 | Reproducibility, Stability |
| mRNA:::rRNA | 52 | 15 | Reproducibility, Stability |
| Mt-rRNA:::tRNA | 45 | 5 | Reproducibility, Stability |
| Mt-tRNA:::Mt-tRNA | 42 | 0 | Reproducibility, Stability |
| Mt-rRNA:::processed-transcript | 40 | 13 | Reproducibility, Stability |
| antisense:::Mt-rRNA | 38 | 6 | Reproducibility, Stability |
| snoRNA:::miRNA | 37 | 0 | Reproducibility, Stability |
| misc-RNA:::Mt-rRNA | 30 | 3 | Reproducibility, Stability |
| snRNA:::snRNA | 28 | 14 | Reproducibility, Stability |
| snoRNA:::known-ncrna | 27 | 2 | Reproducibility, Stability, U3, rRNA |
| IG-C-gene:::mRNA | 27 | 10 | Reproducibility, Stability |
| known-ncrna:::lincRNA | 25 | 18 | Reproducibility, Stability |
| miRNA:::processed-transcript | 25 | 3 | Reproducibility, Stability |
| sense-overlapping::: sense-overlapping | 23 | 3 | Reproducibility, Stability |
| Mt-rRNA:::snRNA | 20 | 2 | Reproducibility, Stability |
| antisense:::processed-transcript | 18 | 0 | Reproducibility, Stability |
| antisense:::tRNA | 14 | 0 | Reproducibility, Stability |
| Mt-tRNA:::tRNA | 14 | 3 | Reproducibility, Stability |
| antisense:::misc-RNA | 13 | 4 | Reproducibility, Stability |
| lincRNA:::sense-intronic | 11 | 5 | Reproducibility, Stability |
| antisense:::Mt-tRNA | 9 | 0 | Reproducibility, Stability |
| Mt-rRNA:::sense-overlapping | 9 | 2 | Reproducibility, Stability |
| Mt-tRNA:::processed-transcript | 9 | 0 | Reproducibility, Stability |
| Mt-rRNA:::rRNA | 9 | 2 | Reproducibility, Stability |
| rRNA:::rRNA | 8 | 6 | Reproducibility, Stability |
| misc-RNA:::processed-transcript | 7 | 0 | Reproducibility, Stability |
| misc-RNA:::tRNA | 7 | 0 | Reproducibility, Stability |
| snoRNA:::IG-C-gene | 7 | 0 | Reproducibility, Stability |
| mRNA:::sense-intronic | 7 | 1 | Reproducibility, Stability |
| miRNA:::Mt-rRNA | 6 | 3 | Reproducibility, Stability |
| mRNA:::TEC | 6 | 0 | Reproducibility, Stability |
| antisense:::miRNA | 6 | 0 | Reproducibility, Stability |
| rRNA:::tRNA | 6 | 0 | Reproducibility, Stability |
| antisense:::rRNA | 5 | 2 | Reproducibility, Stability |
| IG-C-gene:::lincRNA | 5 | 0 | Reproducibility, Stability |
| processed-transcript:::tRNA | 5 | 0 | Reproducibility, Stability |
| Mt-rRNA:::sense-intronic | 5 | 2 | Reproducibility, Stability |
| antisense:::snRNA | 5 | 0 | Reproducibility, Stability |
| lincRNA:::TEC | 4 | 0 | Reproducibility, Stability |
| snoRNA:::TEC | 4 | 0 | Reproducibility, Stability |
| misc-RNA:::snRNA | 4 | 0 | Reproducibility, Stability |
| IG-C-gene:::IG-C-gene | 4 | 4 | Reproducibility, Stability |
| misc-RNA:::Mt-tRNA | 4 | 2 | Reproducibility, Stability |
| TEC:::TEC | 4 | 0 | Reproducibility, Stability |
| known-ncrna:::mRNA | 4 | 0 | Reproducibility, Stability |
| Mt-tRNA:::snRNA | 3 | 0 | Reproducibility, Stability |
| antisense:::TEC | 3 | 0 | Reproducibility, Stability |
| processed-transcript::: sense-overlapping | 3 | 0 | Reproducibility, Stability |
| sense-overlapping:::snRNA | 3 | 0 | Reproducibility, Stability |
| rRNA:::snRNA | 3 | 0 | Reproducibility, Stability |
| snRNA:::tRNA | 2 | 0 | Reproducibility, Stability |
| known-ncrna:::misc-RNA | 2 | 0 | Reproducibility, Stability |
| Mt-tRNA:::rRNA | 2 | 0 | Reproducibility, Stability |
| known-ncrna:::known-ncrna | 2 | 0 | Reproducibility, Stability |
| miRNA:::tRNA | 2 | 0 | Reproducibility, Stability |
| mRNA:::TR-C-gene | 2 | 0 | Reproducibility, Stability |
| antisense:::sense-overlapping | 2 | 0 | Reproducibility, Stability |
| IG-C-gene:::processed-transcript | 1 | 0 | Reproducibility, Stability |
| miRNA:::misc-RNA | 1 | 0 | Reproducibility, Stability |
| sense-overlapping:::tRNA | 1 | 0 | Reproducibility, Stability |
| snoRNA:::sense-intronic | 1 | 0 | Reproducibility, Stability |
| sense-intronic:::TEC | 1 | 0 | Reproducibility, Stability |
| known-ncrna:::tRNA | 1 | 0 | Reproducibility, Stability |
| miRNA:::Mt-tRNA | 1 | 0 | Reproducibility, Stability |
| mRNA:::non-coding | 1 | 0 | Reproducibility, Stability |
| 3prime-overlapping-ncrna::: mRNA | 1 | 0 | Reproducibility, Stability |

#

# Table S2: High confidence methylating hybrids

| Methylation site | snoRNA families | Box | Hybrid count | Source count | Category |
| --- | --- | --- | --- | --- | --- |
| 28S-4588 | SNORD91 | (D’ box) | 1,994 | 16 | Reported |
| 18S-462 | SNORD14 | (D box) | 670 | 12 | Reported |
| 18S-121 | snoZ17 | (D’ box) | 667 | 13 | Reported |
| 18S-27 | SNORD27 | (D’ box) | 653 | 9 | Reported |
| 28S-1858 | SNORD38 | (D’ box) | 435 | 11 | Reported |
| 18S-1490 | SNORD25 | (D’ box) | 427 | 10 | Reported |
| 18S-159 | SNORD45 | (D box) | 417 | 10 | Reported |
| 18S-1288 | SNORD110 | (D’ box) | 416 | 13 | Reported |
| 18S-83 | SNORD14 | (D’ box) | 299 | 11 | Not reported |
| 18S-512 | SNORD70 | (D box) | 277 | 12 | Reported |
| 18S-428 | snoMBII-202 | (D’ box) | 259 | 12 | Reported |
| 18S-1442 | SNORD61 | (D box) | 257 | 9 | Reported |
| 18S-99 | SNORD57 | (D box) | 218 | 9 | Reported |
| 28S-2338 | SNORD24 | (D box) | 194 | 10 | Reported |
| 18S-627 | SNORD65 | (D’ box) | 194 | 9 | Reported |
| 18S-509 | SNORD11B | (D box) | 182 | 9 | Reported |
| 18S-668 | SNORD36 | (D box) | 165 | 2 | Reported |
| 28S-2352 | SNORD24 | (D’ box) | 155 | 10 | Reported |
| 28S-4166 | SNORD31 | (D’ box) | 154 | 9 | Reported |
| 28S-2402 | SNORD143, SNORD144 | (D’ box) | 153 | 10 | Reported |
| 5.8S-75 | SNORD96 | (D box) | 121 | 9 | Reported |
| 18S-116 | SNORD42 | (D box) | 116 | 7 | Reported |
| 18S-1806 | SNORD20 | (D box) | 110 | 9 | Reported |
| 18S-1705 | SNORD43 | (D’ box) | 91 | 3 | Reported |
| 18S-799 | SNORD105, snoU105B | (D box) | 86 | 9 | Reported |
| 28S-2774 | SNORD99 | (D box) | 66 | 10 | Reported |
| 28S-2409 | SNORD5 | (D’ box) | 58 | 9 | Reported |
| 28S-4590 | SNORD72 | (D’ box) | 53 | 2 | Reported |
| 28S-391 | SNORD81 | (D’ box) | 42 | 9 | Reported |
| 18S-1031 | SNORD59 | (D’ box) | 41 | 2 | Reported |
| 28S-2411 | snoZ40 | (D’ box) | 36 | 6 | Reported |
| 18S-47 | SNORD35 | (D box) | 34 | 2 | Not reported |
| 18S-172 | SNORD45 | (D’ box) | 33 | 8 | Reported |
| 28S-2802 | SNORD95 | (D box) | 31 | 7 | Reported |
| 18S-1440 | SNORD125 | (D box) | 30 | 8 | Reported |
| 28S-2811 | SNORD95 | (D’ box) | 30 | 7 | Reported |
| 28S-389 | SNORD26 | (D’ box) | 28 | 7 | Reported |
| 28S-1511 | SNORD51, SNORD33 | (D’ box) | 28 | 7 | Reported |
| 28S-2350 | SNORD24 | (D’ box) | 28 | 2 | Reported |
| 28S-1868 | SNORD48 | (D box) | 23 | 8 | Reported |
| 28S-1313 | SNORD18 | (D’ box) | 21 | 4 | Reported |
| 28S-1327 | SNORND104 | (D’ box) | 17 | 5 | Reported |
| 28S-2824 | SNORD34 | (D’ box) | 17 | 5 | Reported |
| 18S-517 | SNORD56 | (D’ box) | 16 | 3 | Reported |
| 28S-4541 | SNORD63 | (D’ box) | 16 | 3 | Reported |
| 28S-3723 | SNORD87 | (D’ box) | 14 | 4 | Reported |
| 28S-3703 | SNORD36 | (D’ box) | 13 | 1 | Reported |
| 28S-4662 | SNORD15 | (D box) | 9 | 1 | Not reported |
| 28S-3923 | SNORD111 | (D’ box) | 8 | 3 | Reported |
| 18S-683 | SNORD19B | (D box) | 8 | 2 | Reported |
| 28S-3697 | SNORD37 | (D’ box) | 8 | 2 | Reported |
| 18S-189 | snoU83B | (D’ box) | 6 | 1 | Not reported |
| 28S-1310 | SNORD126 | (D’ box) | 5 | 2 | Not reported |
| 28S-3764 | SNORD15 | (D box) | 5 | 4 | Reported |
| 18S-644 | snoU54 | (D box) | 4 | 1 | Reported |
| 28S-4999 | SNORD62 | (D’ box) | 2 | 1 | Not reported |
| 18S-867 | SNORD98 | (D’ box) | 2 | 1 | Reported |
| 28S-3904 | SNORD52 | (D’ box) | 2 | 1 | Reported |
| 28S-879 | SNORD73 | (D’ box) | 1 | 1 | Not reported |
| 18S-174 | SNORD45 | (D’ box) | 1 | 1 | Reported |
| 28S-4522 | SNORD63 | (D box) | 1 | 1 | Reported |

#

# Table S3: Potentially methylating hybrids

| Methylation site | snoRNA families | Box | Hybrid count | | | Source count | | | | Category | | | |  |  |  |
| --- | --- | --- | --- | --- | --- | --- | --- | --- | --- | --- | --- | --- | --- | --- | --- | --- |
| 28S-4588 | SNORD91 | (D’ box) | 1,799 | | | 15 | | | | Reported | | | |  |  |  |
| 18S-468 | snoU83B | (D box) | 904 | | | 14 | | | | Reported | | | |  |  |  |
| 18S-509 | SNORD11, SNORD11B | (D box) | 409 | | | 11 | | | | Reported | | | |  |  |  |
| 18S-412 | U8 | (D box) | 401 | | | 12 | | | | Not reported | | | |  |  |  |
| 18S-116 | SNORD42 | (D box) | 360 | | | 7 | | | | Reported | | | |  |  |  |
| 28S-1612 | snosnR60_Z15 | (D box) | 243 | | | 10 | | | | Reported | | | |  |  |  |
| 28S-2848 | SNORD50 | (D box) | 214 | | | 11 | | | | Reported | | | |  |  |  |
| 18S-484 | SNORD16 | (D’ box) | 212 | | | 7 | | | | Reported | | | |  |  |  |
| 18S-172 | SNORD45 | (D’ box) | 208 | | | 10 | | | | Reported | | | |  |  |  |
| 28S-1747 | SNORD73 | (D’ box) | 195 | | | 8 | | | | Reported | | | |  |  |  |
| 28S-4593 | SNORD78 | (D’ box) | 168 | | | 8 | | | | Reported | | | |  |  |  |
| 28S-3723 | SNORD87 | (D’ box) | 162 | | | 10 | | | | Reported | | | |  |  |  |
| 28S-15 | SNORD47, SNORD91, SNORD36 | (D box) | 151 | | | 3 | | | | Not reported | | | |  |  |  |
| 28S-4506 | SNORD35 | (D’ box) | 136 | | | 9 | | | | Reported | | | |  |  |  |
| 18S-1605 | U8, SNORD11B | (D box) | 124 | | | 4 | | | | Not reported | | | |  |  |  |
| 28S-2863 | SNORD50 | (D’ box) | 124 | | | 10 | | | | Reported | | | |  |  |  |
| 18S-95 | SNORD14 | (D’ box) | 105 | | | 6 | | | | Not reported | | | |  |  |  |
| 28S-2388 | snoMBII-202 | (D box) | 93 | | | 10 | | | | Reported | | | |  |  |  |
| 18S-436 | SNORD100 | (D’ box) | 74 | | | 9 | | | | Reported | | | |  |  |  |
| 18S-1452 | snoU83B, SNORD51 | (D box) | 71 | | | 1 | | | | Not reported | | | |  |  |  |
| 18S-174 | SNORD45 | (D’ box) | 69 | | | 4 | | | | Reported | | | |  |  |  |
| 28S-20 | SNORD43, SCARNA7 | (D box) | 67 | | | 1 | | | | Not reported | | | |  |  |  |
| 28S-21 | snoR38, SNORD16 | (D’ box) | 67 | | | 1 | | | | Not reported | | | |  |  |  |
| 18S-1272 | SNORD66 | (D’ box) | 64 | | | 6 | | | | Reported | | | |  |  |  |
| 28S-4581 | SNORD78 | (D box) | 63 | | | 6 | | | | Not reported | | | |  |  |  |
| 18S-502 | SNORD14 | (D box) | 49 | | | 6 | | | | Not reported | | | |  |  |  |
| 18S-507 | snoU83B | (D box) | 48 | | | 3 | | | | Not reported | | | |  |  |  |
| 18S-1164 | SNORD2, SNORD70 | (D’ box) | 41 | | | 5 | | | | Not reported | | | |  |  |  |
| 28S-1509 | SNORD2 | (D’ box) | 40 | | | 6 | | | | Reported | | | |  |  |  |
| 28S-30 | SNORD43 | (D’ box) | 37 | | | 1 | | | | Not reported | | | |  |  |  |
| 5.8S-14 | SNORD71, SNORD21 | (D’ box) | 37 | | | 6 | | | | Reported | | | |  |  |  |
| 28S-2435 | SNORD2 | (D’ box) | 35 | | | 5 | | | | Not reported | | | |  |  |  |
| 18S-601 | SNORD103 | (D’ box) | 34 | | | 5 | | | | Reported | | | |  |  |  |
| 18S-428 | snoMBII-202 | (D’ box) | 33 | | | 5 | | | | Reported | | | |  |  |  |
| 28S-1858 | SNORD38 | (D’ box) | 30 | | | 7 | | | | Reported | | | |  |  |  |
| 18S-854 | SNORD53_SNORD92 | (D box) | 28 | | | 3 | | | | Not reported | | | |  |  |  |
| 18S-189 | snoU83B | (D’ box) | 26 | | | 4 | | | | Not reported | | | |  |  |  |
| 18S-1433 | SNORD74 | (D’ box) | 25 | | | 2 | | | | Not reported | | | |  |  |  |
| 28S-17 | SNORD77 | (D box) | 25 | | | 1 | | | | Not reported | | | |  |  |  |
| 18S-1610 | SNORD50 | (D’ box) | 24 | | | 1 | | | | Not reported | | | |  |  |  |
| 28S-4560 | SNORD107 | (D box) | 24 | | | 5 | | | | Reported | | | |  |  |  |
| 18S-1425 | SNORD74 | (D box) | 23 | | | 2 | | | | Not reported | | | |  |  |  |
| 28S-19 | SNORD111 | (D box) | 23 | | | 1 | | | | Not reported | | | |  |  |  |
| 18S-1263 | SNORD66 | (D box) | 22 | | | 5 | | | | Not reported | | | |  |  |  |
| 28S-56 | SNORD12 | (D box) | 22 | | | 1 | | | | Not reported | | | |  |  |  |
| 18S-1391 | SNORD28 | (D box) | 22 | | | 1 | | | | Reported | | | |  |  |  |
| 18S-405 | SNORD100 | (D box) | 21 | | | 3 | | | | Not reported | | | |  |  |  |
| 18S-411 | SNORD100 | (D’ box) | 21 | | | 3 | | | | Not reported | | | |  |  |  |
| 18S-1615 | SNORD110 | (D’ box) | 21 | | | 3 | | | | Not reported | | | |  |  |  |
| 18S-1161 | snoMBII-202 | (D’ box) | 21 | | | 2 | | | | Not reported | | | |  |  |  |
| 28S-1456 | U3 | (D box) | 18 | | | 4 | | | | Not reported | | | |  |  |  |
| 18S-1452 | SNORD59, SNORD79 | (D’ box) | 17 | | | 1 | | | | Not reported | | | |  |  |  |
| 28S-3031 | U3 | (D box) | 16 | | | 4 | | | | Not reported | | | |  |  |  |
| 18S-157 | SNORD65 | (D’ box) | 16 | | | 1 | | | | Not reported | | | |  |  |  |
| 28S-4541 | SNORD63 | (D’ box) | 16 | | | 4 | | | | Reported | | | |  |  |  |
| 28S-2197 | U3 | (D box) | 14 | | | 6 | | | | Not reported | | | |  |  |  |
| 28S-4586 | SNORD37 | (D box) | 13 | | | 4 | | | | Not reported | | | |  |  |  |
| 18S-1605 | SNORD12 | (D’ box) | 13 | | | 2 | | | | Not reported | | | |  |  |  |
| 28S-22 | snoR38 | (D’ box) | 13 | | | 1 | | | | Not reported | | | |  |  |  |
| 28S-336 | snoZ17 | (D box) | 13 | | | 1 | | | | Not reported | | | |  |  |  |
| 18S-668 | SNORD36 | (D box) | 13 | | | 1 | | | | Reported | | | |  |  |  |
| 28S-4650 | SNORD11B | (D box) | 12 | | | 3 | | | | Not reported | | | |  |  |  |
| 18S-1170 | snoU83B | (D box) | 12 | | | 2 | | | | Not reported | | | |  |  |  |
| 28S-2347 | SNORD70, SNORD27 | (D box) | 11 | | | 3 | | | | Not reported | | | |  |  |  |
| 28S-26 | SNORD43 | (D’ box) | 11 | | | 1 | | | | Not reported | | | |  |  |  |
| 18S-435 | snoU83B | (D box) | 10 | | | 2 | | | | Not reported | | | |  |  |  |
| 18S-21 | SNORD53_SNORD92 | (D’ box) | 10 | | | 1 | | | | Not reported | | | |  |  |  |
| 18S-504 | SNORD56 | (D box) | 10 | | | 1 | | | | Not reported | | | |  |  |  |
| 28S-44 | SNORD25 | (D box) | 10 | | | 1 | | | | Not reported | | | |  |  |  |
| 28S-54 | SNORD38 | (D’ box) | 10 | | | 1 | | | | Not reported | | | |  |  |  |
| 28S-4522 | SNORD63 | (D box) | 10 | | | 4 | | | | Reported | | | |  |  |  |
| 18S-596 | SNORD30 | (D box) | 9 | | | 3 | | | | Not reported | | | |  |  |  |
| 18S-115 | SNORD31, SNORD22 | (D box) | 9 | | | 2 | | | | Not reported | | | |  |  |  |
| 18S-410 | SNORD36 | (D’ box) | 9 | | | 2 | | | | Not reported | | | |  |  |  |
| 18S-856 | SNORD35 | (D’ box) | 9 | | | 2 | | | | Not reported | | | |  |  |  |
| 28S-13 | SNORD66 | (D’ box) | 9 | | | 2 | | | | Not reported | | | |  |  |  |
| 18S-113 | SNORD2 | (D box) | 9 | | | 1 | | | | Not reported | | | |  |  |  |
| 18S-1525 | SNORD10 | (D’ box) | 9 | | | 1 | | | | Not reported | | | |  |  |  |
| 28S-3697 | SNORD37 | (D’ box) | 9 | | | 3 | | | | Reported | | | |  |  |  |
| 18S-462 | SNORD14 | (D box) | 9 | | | 2 | | | | Reported | | | |  |  |  |
| 18S-1490 | SNORD25 | (D’ box) | 9 | | | 2 | | | | Reported | | | |  |  |  |
| 28S-4592 | SNORD57 | (D’ box) | 8 | | | 4 | | | | Not reported | | | |  |  |  |
| 28S-2796 | SNORD100 | (D box) | 8 | | | 2 | | | | Not reported | | | |  |  |  |
| 18S-127 | SNORD43 | (D’ box) | 8 | | | 1 | | | | Not reported | | | |  |  |  |
| 18S-1577 | snoU13 | (D box) | 8 | | | 1 | | | | Not reported | | | |  |  |  |
| 18S-1739 | SNORD103, SNORD36 | (D’ box) | 8 | | | 1 | | | | Not reported | | | |  |  |  |
| 28S-14 | SNORD50, SNORD86 | (D box) | 8 | | | 1 | | | | Not reported | | | |  |  |  |
| 18S-861 | snoU83B | (D box) | 7 | | | 4 | | | | Not reported | | | |  |  |  |
| 28S-1086 | U3 | (D box) | 7 | | | 2 | | | | Not reported | | | |  |  |  |
| 28S-4649 | SNORD11B | (D box) | 7 | | | 2 | | | | Not reported | | | |  |  |  |
| 18S-10 | SNORD25 | (D box) | 7 | | | 1 | | | | Not reported | | | |  |  |  |
| 18S-22 | SNORD24 | (D’ box) | 7 | | | 1 | | | | Not reported | | | |  |  |  |
| 18S-128 | snoU83B | (D’ box) | 7 | | | 1 | | | | Not reported | | | |  |  |  |
| 18S-562 | SNORD103 | (D box) | 7 | | | 1 | | | | Not reported | | | |  |  |  |
| 18S-993 | SNORD59, SNORD30 | (D’ box) | 7 | | | 1 | | | | Not reported | | | |  |  |  |
| 18S-1210 | U8 | (D box) | 7 | | | 1 | | | | Not reported | | | |  |  |  |
| 18S-1510 | SNORD58 | (D’ box) | 7 | | | 1 | | | | Not reported | | | |  |  |  |
| 18S-1519 | snoU83B | (D’ box) | 7 | | | 1 | | | | Not reported | | | |  |  |  |
| 28S-60 | SNORD59 | (D’ box) | 7 | | | 1 | | | | Not reported | | | |  |  |  |
| 28S-116 | SNORD16 | (D’ box) | 7 | | | 1 | | | | Not reported | | | |  |  |  |
| 18S-27 | SNORD27 | (D’ box) | 7 | | | 1 | | | | Reported | | | |  |  |  |
| 18S-34 | SNORD28 | (D’ box) | 7 | | | 1 | | | | Reported | | | |  |  |  |
| 18S-1163 | SNORD90 | (D box) | 6 | | | 3 | | | | Not reported | | | |  |  |  |
| 28S-1716 | SNORD39 | (D’ box) | 6 | | | 3 | | | | Not reported | | | |  |  |  |
| 18S-126 | SNORD96 | (D box) | 6 | | | 2 | | | | Not reported | | | |  |  |  |
| 18S-963 | SNORD36 | (D’ box) | 6 | | | 2 | | | | Not reported | | | |  |  |  |
| 18S-1512 | SNORD14 | (D box) | 6 | | | 2 | | | | Not reported | | | |  |  |  |
| 18S-1822 | snoU13 | (D box) | 6 | | | 2 | | | | Not reported | | | |  |  |  |
| 28S-4494 | SNORD35 | (D box) | 6 | | | 2 | | | | Not reported | | | |  |  |  |
| 18S-409 | snoZ17 | (D box) | 6 | | | 1 | | | | Not reported | | | |  |  |  |
| 18S-1201 | SNORD110 | (D’ box) | 6 | | | 1 | | | | Not reported | | | |  |  |  |
| 28S-2382 | SNORD37 | (D’ box) | 6 | | | 1 | | | | Not reported | | | |  |  |  |
| 28S-4964 | SNORD107 | (D’ box) | 6 | | | 1 | | | | Not reported | | | |  |  |  |
| 28S-5001 | SNORD24 | (D box) | 6 | | | 1 | | | | Not reported | | | |  |  |  |
| 18S-517 | SNORD56 | (D’ box) | 6 | | | 1 | | | | Reported | | | |  |  |  |
| 18S-619 | SNORD53_SNORD92 | (D box) | 5 | | | 2 | | | | Not reported | | | |  |  |  |
| 18S-1137 | snoMBII-202 | (D’ box) | 5 | | | 2 | | | | Not reported | | | |  |  |  |
| 28S-805 | U3 | (D box) | 5 | | | 2 | | | | Not reported | | | |  |  |  |
| 18S-90 | SNORD16 | (D’ box) | 5 | | | 1 | | | | Not reported | | | |  |  |  |
| 18S-830 | SNORD18 | (D’ box) | 5 | | | 1 | | | | Not reported | | | |  |  |  |
| 18S-1598 | SNORD33 | (D box) | 5 | | | 1 | | | | Not reported | | | |  |  |  |
| 18S-1702 | SNORD65 | (D box) | 5 | | | 1 | | | | Not reported | | | |  |  |  |
| 18S-1730 | SNORD65 | (D’ box) | 5 | | | 1 | | | | Not reported | | | |  |  |  |
| 28S-13 | SNORD66 | (D box) | 5 | | | 1 | | | | Not reported | | | |  |  |  |
| 28S-14 | SNORD95 | (D’ box) | 5 | | | 1 | | | | Not reported | | | |  |  |  |
| 28S-2333 | SNORD24 | (D’ box) | 4 | | | 3 | | | | Not reported | | | |  |  |  |
| 28S-2389 | SNORD30 | (D’ box) | 4 | | | 3 | | | | Not reported | | | |  |  |  |
| 28S-4044 | U3 | (D box) | 4 | | | 2 | | | | Not reported | | | |  |  |  |
| 18S-79 | SNORD70 | (D box) | 4 | | | 1 | | | | Not reported | | | |  |  |  |
| 18S-646 | SNORD58 | (D’ box) | 4 | | | 1 | | | | Not reported | | | |  |  |  |
| 18S-828 | SNORD35 | (D box) | 4 | | | 1 | | | | Not reported | | | |  |  |  |
| 18S-1450 | SNORD103 | (D’ box) | 4 | | | 1 | | | | Not reported | | | |  |  |  |
| 18S-1473 | SNORD2, SNORD51 | (D’ box) | 4 | | | 1 | | | | Not reported | | | |  |  |  |
| 18S-1546 | snoU54 | (D box) | 4 | | | 1 | | | | Not reported | | | |  |  |  |
| 18S-1674 | SNORD110 | (D’ box) | 4 | | | 1 | | | | Not reported | | | |  |  |  |
| 28S-723 | snoU83B | (D box) | 4 | | | 1 | | | | Not reported | | | |  |  |  |
| 28S-1604 | SNORD83 | (D box) | 4 | | | 1 | | | | Not reported | | | |  |  |  |
| 28S-2414 | SNORD2 | (D box) | 4 | | | 1 | | | | Not reported | | | |  |  |  |
| 28S-2430 | SNORD58 | (D’ box) | 4 | | | 1 | | | | Not reported | | | |  |  |  |
| 28S-2502 | SNORD81, SNORD26 | (D’ box) | 4 | | | 1 | | | | Not reported | | | |  |  |  |
| 28S-2514 | SNORD69, SNORD57 | (D’ box) | 4 | | | 1 | | | | Not reported | | | |  |  |  |
| 28S-4718 | SNORD39 | (D box) | 4 | | | 1 | | | | Not reported | | | |  |  |  |
| 28S-5026 | snoU54 | (D’ box) | 4 | | | 1 | | | | Not reported | | | |  |  |  |
| 18S-121 | SNORND104 | (D box) | 4 | | | 2 | | | | Reported | | | |  |  |  |
| 18S-683 | SNORD19 | (D box) | 4 | | | 2 | | | | Reported | | | |  |  |  |
| 18S-1031 | SNORD59 | (D’ box) | 4 | | | 1 | | | | Reported | | | |  |  |  |
| 18S-599 | snoU83B | (D box) | 3 | | | 2 | | | | Not reported | | | |  |  |  |
| 28S-2413 | snoMBII-202 | (D’ box) | 3 | | | 2 | | | | Not reported | | | |  |  |  |
| 28S-4767 | U3 | (D box) | 3 | | | 2 | | | | Not reported | | | |  |  |  |
| 18S-43 | SNORD42 | (D’ box) | 3 | | | 1 | | | | Not reported | | | |  |  |  |
| 18S-84 | SNORD59, SNORD126 | (D’ box) | 3 | | | 1 | | | | Not reported | | | |  |  |  |
| 18S-104 | SNORD19 | (D’ box) | 3 | | | 1 | | | | Not reported | | | |  |  |  |
| 18S-114 | SNORD79 | (D’ box) | 3 | | | 1 | | | | Not reported | | | |  |  |  |
| 18S-421 | SNORD58 | (D’ box) | 3 | | | 1 | | | | Not reported | | | |  |  |  |
| 18S-1285 | SNORD69 | (D box) | 3 | | | 1 | | | | Not reported | | | |  |  |  |
| 18S-1421 | SNORD44 | (D box) | 3 | | | 1 | | | | Not reported | | | |  |  |  |
| 18S-1430 | SNORD44 | (D’ box) | 3 | | | 1 | | | | Not reported | | | |  |  |  |
| 18S-1435 | SNORD43 | (D’ box) | 3 | | | 1 | | | | Not reported | | | |  |  |  |
| 18S-1449 | SNORD60 | (D’ box) | 3 | | | 1 | | | | Not reported | | | |  |  |  |
| 18S-1485 | SNORD34 | (D’ box) | 3 | | | 1 | | | | Not reported | | | |  |  |  |
| 18S-1731 | SNORD30 | (D box) | 3 | | | 1 | | | | Not reported | | | |  |  |  |
| 18S-1735 | SNORD103 | (D box) | 3 | | | 1 | | | | Not reported | | | |  |  |  |
| ITS2-461 | SNORD12 | (D’ box) | 3 | | | 1 | | | | Not reported | | | |  |  |  |
| 28S-2221 | U3 | (D box) | 3 | | | 1 | | | | Not reported | | | |  |  |  |
| 28S-2226 | U3 | (D box) | 3 | | | 1 | | | | Not reported | | | |  |  |  |
| 28S-2496 | SNORD83 | (D’ box) | 3 | | | 1 | | | | Not reported | | | |  |  |  |
| 28S-2807 | SNORD28 | (D box) | 3 | | | 1 | | | | Not reported | | | |  |  |  |
| 28S-2903 | SNORD31 | (D’ box) | 3 | | | 1 | | | | Not reported | | | |  |  |  |
| 28S-4607 | SNORD121A | (D’ box) | 3 | | | 2 | | | | Reported | | | |  |  |  |
| 18S-99 | SNORD57 | (D box) | 3 | | | 1 | | | | Reported | | | |  |  |  |
| 18S-683 | SNORD19B | (D’ box) | 3 | | | 1 | | | | Reported | | | |  |  |  |
| 18S-118 | SNORND104 | (D box) | 2 | | | 2 | | | | Not reported | | | |  |  |  |
| 28S-554 | U3 | (D box) | 2 | | | 2 | | | | Not reported | | | |  |  |  |
| 28S-2898 | SNORD29 | (D’ box) | 2 | | | 2 | | | | Not reported | | | |  |  |  |
| 18S-130 | SNORD2 | (D’ box) | 2 | | | 1 | | | | Not reported | | | |  |  |  |
| 18S-144 | SNORND104 | (D’ box) | 2 | | | 1 | | | | Not reported | | | |  |  |  |
| 18S-389 | snoU6-53 | (D’ box) | 2 | | | 1 | | | | Not reported | | | |  |  |  |
| 18S-416 | SNORD65 | (D box) | 2 | | | 1 | | | | Not reported | | | |  |  |  |
| 18S-591 | SNORD42 | (D box) | 2 | | | 1 | | | | Not reported | | | |  |  |  |
| 18S-612 | SNORD43, SNORD41 | (D box) | 2 | | | 1 | | | | Not reported | | | |  |  |  |
| 18S-613 | SNORD58 | (D’ box) | 2 | | | 1 | | | | Not reported | | | |  |  |  |
| 18S-796 | SNORD25 | (D’ box) | 2 | | | 1 | | | | Not reported | | | |  |  |  |
| 18S-806 | SNORD83 | (D’ box) | 2 | | | 1 | | | | Not reported | | | |  |  |  |
| 18S-817 | SNORD2 | (D box) | 2 | | | 1 | | | | Not reported | | | |  |  |  |
| 18S-836 | SNORD2 | (D’ box) | 2 | | | 1 | | | | Not reported | | | |  |  |  |
| 18S-980 | SNORD16 | (D’ box) | 2 | | | 1 | | | | Not reported | | | |  |  |  |
| 18S-1012 | SNORD103 | (D box) | 2 | | | 1 | | | | Not reported | | | |  |  |  |
| 18S-1048 | SNORD50 | (D’ box) | 2 | | | 1 | | | | Not reported | | | |  |  |  |
| 18S-1128 | SNORD50 | (D box) | 2 | | | 1 | | | | Not reported | | | |  |  |  |
| 18S-1140 | SNORD50 | (D’ box) | 2 | | | 1 | | | | Not reported | | | |  |  |  |
| 18S-1199 | snoU83B | (D box) | 2 | | | 1 | | | | Not reported | | | |  |  |  |
| 18S-1261 | SNORD65 | (D box) | 2 | | | 1 | | | | Not reported | | | |  |  |  |
| 18S-1284 | SNORD38 | (D’ box) | 2 | | | 1 | | | | Not reported | | | |  |  |  |
| 18S-1289 | SNORD20 | (D box) | 2 | | | 1 | | | | Not reported | | | |  |  |  |
| 18S-1412 | U3 | (D box) | 2 | | | 1 | | | | Not reported | | | |  |  |  |
| 18S-1477 | SNORD42, SNORD110 | (D’ box) | 2 | | | 1 | | | | Not reported | | | |  |  |  |
| 18S-1518 | SNORD48 | (D box) | 2 | | | 1 | | | | Not reported | | | |  |  |  |
| 18S-1520 | SNORD91 | (D box) | 2 | | | 1 | | | | Not reported | | | |  |  |  |
| 18S-1549 | SNORD126 | (D box) | 2 | | | 1 | | | | Not reported | | | |  |  |  |
| 18S-1570 | SNORD126 | (D’ box) | 2 | | | 1 | | | | Not reported | | | |  |  |  |
| 18S-1571 | snoZ40 | (D box) | 2 | | | 1 | | | | Not reported | | | |  |  |  |
| 18S-1584 | snoZ40 | (D’ box) | 2 | | | 1 | | | | Not reported | | | |  |  |  |
| 18S-1596 | SNORD142;ZL68 | (D’ box) | 2 | | | 1 | | | | Not reported | | | |  |  |  |
| 18S-1737 | SNORD82 | (D’ box) | 2 | | | 1 | | | | Not reported | | | |  |  |  |
| 18S-1738 | SNORD100, SNORD36 | (D’ box) | 2 | | | 1 | | | | Not reported | | | |  |  |  |
| 18S-1857 | SNORD50 | (D’ box) | 2 | | | 1 | | | | Not reported | | | |  |  |  |
| 5.8S-12 | SNORD36 | (D box) | 2 | | | 1 | | | | Not reported | | | |  |  |  |
| 5.8S-136 | snoU83B | (D’ box) | 2 | | | 1 | | | | Not reported | | | |  |  |  |
| 28S-2 | SNORD166 | (D box) | 2 | | | 1 | | | | Not reported | | | |  |  |  |
| 28S-53 | SNORD125 | (D box) | 2 | | | 1 | | | | Not reported | | | |  |  |  |
| 28S-57 | snoU105B | (D’ box) | 2 | | | 1 | | | | Not reported | | | |  |  |  |
| 28S-1362 | SNORD65 | (D box) | 2 | | | 1 | | | | Not reported | | | |  |  |  |
| 28S-1382 | SNORD65 | (D’ box) | 2 | | | 1 | | | | Not reported | | | |  |  |  |
| 28S-2394 | SNORD62 | (D’ box) | 2 | | | 1 | | | | Not reported | | | |  |  |  |
| 28S-2699 | SNORD31 | (D’ box) | 2 | | | 1 | | | | Not reported | | | |  |  |  |
| 28S-2739 | SNORD12 | (D’ box) | 2 | | | 1 | | | | Not reported | | | |  |  |  |
| 28S-2823 | SNORD103 | (D box) | 2 | | | 1 | | | | Not reported | | | |  |  |  |
| 28S-2871 | snoR38 | (D box) | 2 | | | 1 | | | | Not reported | | | |  |  |  |
| 28S-3596 | SNORD18 | (D’ box) | 2 | | | 1 | | | | Not reported | | | |  |  |  |
| 28S-4630 | SNORD73 | (D’ box) | 2 | | | 1 | | | | Not reported | | | |  |  |  |
| 18S-121 | snoZ17, SNORD71 | (D’ box) | 2 | | | | 2 | | | | Reported | | | |  |  |
| 28S-2802 | SNORD95 | (D box) | 2 | | | | 2 | | | | Reported | | | |  |  |
| 18S-627 | SNORD65 | (D’ box) | 2 | | | | 1 | | | | Reported | | | |  |  |
| 18S-866 | SNORD34 | (D’ box) | 2 | | | | 1 | | | | Reported | | | |  |  |
| 5.8S-14 | SNORD34 | (D box) | 2 | | | | 1 | | | | Reported | | | |  |  |
| 5.8S-69 | SNORD31 | (D box) | 2 | | | | 1 | | | | Reported | | | |  |  |
| 28S-2409 | SNORD53_SNORD92 | (D’ box) | 2 | | | | 1 | | | | Reported | | | |  |  |
| 18S-43 | snoR38 | (D box) | 1 | | | | 1 | | | | Not reported | | | |  |  |
| 18S-47 | SNORD35 | (D box) | 1 | | | | 1 | | | | Not reported | | | |  |  |
| 18S-70 | snoR38 | (D’ box) | 1 | | | | 1 | | | | Not reported | | | |  |  |
| 18S-83 | SNORD14 | (D’ box) | 1 | | | | 1 | | | | Not reported | | | |  |  |
| 18S-102 | SNORD36 | (D box) | 1 | | | | 1 | | | | Not reported | | | |  |  |
| 18S-110 | SNORD135;ZL2 | (D box) | 1 | | | | 1 | | | | Not reported | | | |  |  |
| 18S-113 | SNORD27 | (D’ box) | 1 | | | | 1 | | | | Not reported | | | |  |  |
| 18S-114 | snoR38 | (D box) | 1 | | | | 1 | | | | Not reported | | | |  |  |
| 18S-127 | U3 | (D box) | 1 | | | | 1 | | | | Not reported | | | |  |  |
| 18S-205 | SNORD36 | (D’ box) | 1 | | | | 1 | | | | Not reported | | | |  |  |
| 18S-379 | SNORND104 | (D’ box) | 1 | | | | 1 | | | | Not reported | | | |  |  |
| 18S-396 | SNORD65 | (D’ box) | 1 | | | | 1 | | | | Not reported | | | |  |  |
| 18S-408 | SNORD48 | (D’ box) | 1 | | | | 1 | | | | Not reported | | | |  |  |
| 18S-411 | U8 | (D box) | 1 | | | | 1 | | | | Not reported | | | |  |  |
| 18S-417 | SNORD30 | (D box) | 1 | | | | 1 | | | | Not reported | | | |  |  |
| 18S-430 | SNORND104 | (D box) | 1 | | | | 1 | | | | Not reported | | | |  |  |
| 18S-433 | SNORD30 | (D’ box) | 1 | | | | 1 | | | | Not reported | | | |  |  |
| 18S-441 | SNORD35 | (D’ box) | 1 | | | | 1 | | | | Not reported | | | |  |  |
| 18S-443 | snoU83B | (D’ box) | 1 | | | | 1 | | | | Not reported | | | |  |  |
| 18S-451 | SNORD95 | (D box) | 1 | | | | 1 | | | | Not reported | | | |  |  |
| 18S-453 | SNORND104 | (D’ box) | 1 | | | | 1 | | | | Not reported | | | |  |  |
| 18S-461 | SNORD42 | (D box) | 1 | | | | 1 | | | | Not reported | | | |  |  |
| 18S-467 | SNORD38 | (D box) | 1 | | | | 1 | | | | Not reported | | | |  |  |
| 18S-471 | snoZ40 | (D box) | 1 | | | | 1 | | | | Not reported | | | |  |  |
| 18S-476 | SNORD38 | (D’ box) | 1 | | | | 1 | | | | Not reported | | | |  |  |
| 18S-477 | snoZ40 | (D’ box) | | | 1 | | | | 1 | | | | Not reported | | | |
| 18S-515 | SNORD2 | (D box) | | 1 | | | | 1 | | | | Not reported | | | |  |
| 18S-592 | SNORD35 | (D’ box) | | 1 | | | | 1 | | | | Not reported | | | |  |
| 18S-599 | SNORD14 | (D’ box) | | 1 | | | | 1 | | | | Not reported | | | |  |
| 18S-600 | U8 | (D box) | | 1 | | | | 1 | | | | Not reported | | | |  |
| 18S-613 | SNORD172;ZL24 | (D box) | | 1 | | | | 1 | | | | Not reported | | | |  |
| 18S-617 | SNORD30 | (D’ box) | | 1 | | | | 1 | | | | Not reported | | | |  |
| 18S-621 | snoZ17 | (D box) | | 1 | | | | 1 | | | | Not reported | | | |  |
| 18S-623 | SNORD30 | (D’ box) | | 1 | | | | 1 | | | | Not reported | | | |  |
| 18S-702 | SNORD19 | (D’ box) | | 1 | | | | 1 | | | | Not reported | | | |  |
| 18S-788 | snoU13 | (D’ box) | | 1 | | | | 1 | | | | Not reported | | | |  |
| 18S-818 | SNORD44 | (D box) | | 1 | | | | 1 | | | | Not reported | | | |  |
| 18S-819 | SNORD60 | (D’ box) | | 1 | | | | 1 | | | | Not reported | | | |  |
| 18S-827 | SNORD95 | (D box) | | 1 | | | | 1 | | | | Not reported | | | |  |
| 18S-829 | SNORD44 | (D’ box) | | 1 | | | | 1 | | | | Not reported | | | |  |
| 18S-893 | SNORD63 | (D box) | | 1 | | | | 1 | | | | Not reported | | | |  |
| 18S-961 | SNORD58 | (D’ box) | | 1 | | | | 1 | | | | Not reported | | | |  |
| 18S-969 | SNORD59 | (D box) | | 1 | | | | 1 | | | | Not reported | | | |  |
| 18S-972 | snoU83B | (D box) | | 1 | | | | 1 | | | | Not reported | | | |  |
| 18S-983 | SNORD18 | (D’ box) | | 1 | | | | 1 | | | | Not reported | | | |  |
| 18S-1052 | SNORD59 | (D’ box) | | 1 | | | | 1 | | | | Not reported | | | |  |
| 18S-1163 | SNORD83 | (D’ box) | | 1 | | | | 1 | | | | Not reported | | | |  |
| 18S-1166 | snoU83B | (D box) | | 1 | | | | 1 | | | | Not reported | | | |  |
| 18S-1168 | SNORD36 | (D’ box) | | 1 | | | | 1 | | | | Not reported | | | |  |
| 18S-1184 | snoU13 | (D’ box) | | 1 | | | | 1 | | | | Not reported | | | |  |
| 18S-1229 | SNORD67 | (D’ box) | | 1 | | | | 1 | | | | Not reported | | | |  |
| 18S-1231 | SNORD48 | (D box) | | 1 | | | | 1 | | | | Not reported | | | |  |
| 18S-1274 | SNORD50 | (D’ box) | | 1 | | | | 1 | | | | Not reported | | | |  |
| 18S-1423 | U3 | (D box) | | 1 | | | | 1 | | | | Not reported | | | |  |
| 18S-1441 | SNORD42 | (D box) | | 1 | | | | 1 | | | | Not reported | | | |  |
| 18S-1453 | SNORD124 | (D box) | | 1 | | | | 1 | | | | Not reported | | | |  |
| 18S-1511 | snoMBII-202 | (D’ box) | | 1 | | | | 1 | | | | Not reported | | | |  |
| 18S-1546 | snoU13 | (D’ box) | 1 | | | 1 | | | | Not reported | | | |  |  |  |
| 18S-1573 | SNORD110 | (D box) | 1 | | | 1 | | | | Not reported | | | |  |  |  |
| 18S-1574 | snoZ17 | (D box) | 1 | | | 1 | | | | Not reported | | | |  |  |  |
| 18S-1575 | snoR38 | (D’ box) | 1 | | | 1 | | | | Not reported | | | |  |  |  |
| 18S-1616 | SNORD59 | (D box) | 1 | | | 1 | | | | Not reported | | | |  |  |  |
| 18S-1667 | SNORD35 | (D box) | 1 | | | 1 | | | | Not reported | | | |  |  |  |
| 18S-1706 | SNORD35 | (D’ box) | 1 | | | 1 | | | | Not reported | | | |  |  |  |
| 18S-1725 | snoU13 | (D’ box) | 1 | | | 1 | | | | Not reported | | | |  |  |  |
| 18S-1730 | SNORD67 | (D box) | 1 | | | 1 | | | | Not reported | | | |  |  |  |
| 18S-1733 | SNORD57 | (D box) | 1 | | | 1 | | | | Not reported | | | |  |  |  |
| 18S-1734 | SNORD100 | (D box) | 1 | | | 1 | | | | Not reported | | | |  |  |  |
| 18S-1736 | SNORD2 | (D box) | 1 | | | 1 | | | | Not reported | | | |  |  |  |
| 18S-1740 | SNORD43 | (D’ box) | 1 | | | 1 | | | | Not reported | | | |  |  |  |
| 18S-1758 | SNORD133;ZL142 | (D box) | 1 | | | 1 | | | | Not reported | | | |  |  |  |
| 5.8S-29 | SNORD27 | (D’ box) | 1 | | | 1 | | | | Not reported | | | |  |  |  |
| 5.8S-41 | SNORD36 | (D’ box) | 1 | | | 1 | | | | Not reported | | | |  |  |  |
| 5.8S-50 | SNORND104 | (D’ box) | 1 | | | 1 | | | | Not reported | | | |  |  |  |
| 5.8S-108 | SNORD38 | (D box) | 1 | | | 1 | | | | Not reported | | | |  |  |  |
| 5.8S-114 | SNORD38 | (D’ box) | 1 | | | 1 | | | | Not reported | | | |  |  |  |
| 5.8S-116 | SNORD65 | (D box) | 1 | | | 1 | | | | Not reported | | | |  |  |  |
| 28S-23 | SNORD49 | (D’ box) | 1 | | | 1 | | | | Not reported | | | |  |  |  |
| 28S-28 | SNORD88 | (D’ box) | 1 | | | 1 | | | | Not reported | | | |  |  |  |
| 28S-29 | SNORD166 | (D’ box) | 1 | | | 1 | | | | Not reported | | | |  |  |  |
| 28S-69 | SNORD12 | (D box) | 1 | | | 1 | | | | Not reported | | | |  |  |  |
| 28S-75 | snoR38 | (D box) | 1 | | | 1 | | | | Not reported | | | |  |  |  |
| 28S-103 | snoR38 | (D’ box) | 1 | | | 1 | | | | Not reported | | | |  |  |  |
| 28S-208 | SNORD47 | (D box) | 1 | | | 1 | | | | Not reported | | | |  |  |  |
| 28S-378 | SNORD111 | (D’ box) | 1 | | | 1 | | | | Not reported | | | |  |  |  |
| 28S-383 | SNORD26 | (D box) | 1 | | | 1 | | | | Not reported | | | |  |  |  |
| 28S-1419 | SNORD2 | (D’ box) | 1 | | | 1 | | | | Not reported | | | |  |  |  |
| 28S-1576 | SNORND104 | (D’ box) | 1 | | | 1 | | | | Not reported | | | |  |  |  |
| 28S-1722 | SNORD65 | (D’ box) | 1 | | | 1 | | | | Not reported | | | |  |  |  |
| 28S-1726 | SNORD25 | (D’ box) | 1 | | | 1 | | | | Not reported | | | |  |  |  |
| 28S-1831 | SNORD103 | (D box) | 1 | | | 1 | | | | Not reported | | | |  |  |  |
| 28S-1932 | SNORD60 | (D’ box) | 1 | | | 1 | | | | Not reported | | | |  |  |  |
| 28S-2215 | U3 | (D box) | 1 | | | 1 | | | | Not reported | | | |  |  |  |
| 28S-2330 | SNORD33 | (D box) | 1 | | | 1 | | | | Not reported | | | |  |  |  |
| 28S-2335 | SNORD24 | (D box) | 1 | | | 1 | | | | Not reported | | | |  |  |  |
| 28S-2344 | SNORD24 | (D’ box) | 1 | | | 1 | | | | Not reported | | | |  |  |  |
| 28S-2367 | SNORD33 | (D’ box) | 1 | | | 1 | | | | Not reported | | | |  |  |  |
| 28S-2379 | SNORD65 | (D box) | 1 | | | 1 | | | | Not reported | | | |  |  |  |
| 28S-2382 | SNORD12 | (D box) | 1 | | | 1 | | | | Not reported | | | |  |  |  |
| 28S-2391 | SNORD30 | (D’ box) | 1 | | | 1 | | | | Not reported | | | |  |  |  |
| 28S-2392 | SNORD51 | (D box) | 1 | | | 1 | | | | Not reported | | | |  |  |  |
| 28S-2410 | SNORD36 | (D box) | 1 | | | 1 | | | | Not reported | | | |  |  |  |
| 28S-2416 | SNORD30 | (D’ box) | 1 | | | 1 | | | | Not reported | | | |  |  |  |
| 28S-2492 | SNORD50 | (D box) | 1 | | | 1 | | | | Not reported | | | |  |  |  |
| 28S-2828 | SNORD60 | (D’ box) | 1 | | | 1 | | | | Not reported | | | |  |  |  |
| 28S-2831 | SNORD27 | (D box) | 1 | | | 1 | | | | Not reported | | | |  |  |  |
| 28S-2844 | SNORD16 | (D’ box) | 1 | | | 1 | | | | Not reported | | | |  |  |  |
| 28S-2887 | SNORD50 | (D box) | 1 | | | 1 | | | | Not reported | | | |  |  |  |
| 28S-2897 | SNORD50 | (D’ box) | 1 | | | 1 | | | | Not reported | | | |  |  |  |
| 28S-3446 | U3 | (D box) | 1 | | | 1 | | | | Not reported | | | |  |  |  |
| 28S-3584 | SNORD14 | (D box) | 1 | | | 1 | | | | Not reported | | | |  |  |  |
| 28S-3615 | SNORD35 | (D’ box) | 1 | | | 1 | | | | Not reported | | | |  |  |  |
| 28S-3626 | SNORD51 | (D box) | 1 | | | 1 | | | | Not reported | | | |  |  |  |
| 28S-3641 | snoU83B | (D box) | 1 | | | 1 | | | | Not reported | | | |  |  |  |
| 28S-3659 | snoU83B | (D’ box) | 1 | | | 1 | | | | Not reported | | | |  |  |  |
| 28S-3734 | U8 | (D box) | 1 | | | 1 | | | | Not reported | | | |  |  |  |
| 28S-4459 | snoU13 | (D’ box) | 1 | | | 1 | | | | Not reported | | | |  |  |  |
| 28S-4486 | SNORD14 | (D’ box) | 1 | | | 1 | | | | Not reported | | | |  |  |  |
| 28S-4495 | SNORD35 | (D’ box) | 1 | | | 1 | | | | Not reported | | | |  |  |  |
| 28S-4499 | SNORD91 | (D’ box) | 1 | | | 1 | | | | Not reported | | | |  |  |  |
| 28S-4572 | SNORD30 | (D’ box) | 1 | | | 1 | | | | Not reported | | | |  |  |  |
| 28S-4576 | SNORD100 | (D’ box) | 1 | | | 1 | | | | Not reported | | | |  |  |  |
| 28S-4589 | SNORD121A | (D box) | 1 | | | 1 | | | | Not reported | | | |  |  |  |
| 28S-4596 | SNORD110 | (D box) | 1 | | | 1 | | | | Not reported | | | |  |  |  |
| 28S-4603 | SNORD105 | (D’ box) | 1 | | | 1 | | | | Not reported | | | |  |  |  |
| 28S-4652 | snoZ17 | (D box) | 1 | | | 1 | | | | Not reported | | | |  |  |  |
| 28S-4849 | SNORD58 | (D’ box) | 1 | | | 1 | | | | Not reported | | | |  |  |  |
| 28S-4958 | SNORD25 | (D’ box) | 1 | | | 1 | | | | Not reported | | | |  |  |  |
| 28S-4979 | SNORD38 | (D’ box) | 1 | | | 1 | | | | Not reported | | | |  |  |  |
| 28S-4994 | SNORD48 | (D box) | 1 | | | 1 | | | | Not reported | | | |  |  |  |
| 28S-4996 | snoU54 | (D box) | 1 | | | 1 | | | | Not reported | | | |  |  |  |
| 28S-4996 | SNORD30 | (D’ box) | 1 | | | 1 | | | | Not reported | | | |  |  |  |
| 28S-5003 | SNORD96 | (D’ box) | 1 | | | 1 | | | | Not reported | | | |  |  |  |
| 18S-27 | SNORD15 | (D box) | 1 | | | 1 | | | | Reported | | | |  |  |  |
| 18S-644 | snoU54 | (D box) | 1 | | | 1 | | | | Reported | | | |  |  |  |
| 18S-867 | SNORD98 | (D’ box) | 1 | | | 1 | | | | Reported | | | |  |  |  |
| 18S-1172 | SNORD31 | (D box) | 1 | | | 1 | | | | Reported | | | |  |  |  |
| 18S-1288 | SNORD110 | (D’ box) | 1 | | | 1 | | | | Reported | | | |  |  |  |
| 18S-1447 | snoU89 | (D box) | 1 | | | 1 | | | | Reported | | | |  |  |  |
| 18S-1536 | snoU89 | (D box) | 1 | | | 1 | | | | Reported | | | |  |  |  |
| 18S-1668 | SNORD67 | (D box) | 1 | | | 1 | | | | Reported | | | |  |  |  |
| 18S-1668 | SNORD94 | (D’ box) | 1 | | | 1 | | | | Reported | | | |  |  |  |
| 28S-389 | SNORD26 | (D’ box) | 1 | | | 1 | | | | Reported | | | |  |  |  |
| 28S-2791 | SNORD35 | (D’ box) | 1 | | | 1 | | | | Reported | | | |  |  |  |
| 28S-3764 | SNORD15 | (D box) | 1 | | | 1 | | | | Reported | | | |  |  |  |
| 28S-4032 | SNORD75 | (D’ box) | 1 | | | 1 | | | | Reported | | | |  |  |  |

#

# Table S4: Ancillary hybrid counts

| Interaction | Hybrid count | Experiment count |
| --- | --- | --- |
| SNORD25 : 18S-10 | 35 | 2 |
| SNORD31 : 5.8S-69 | 23 | 1 |
| SNORD48 : 18S-1518 | 19 | 1 |
| SNORD59 : 28S-60 | 19 | 1 |
| SNORD2 : 18S-113 | 17 | 1 |
| SNORD58 : 18S-421 | 17 | 1 |
| SNORD20 : 18S-1804 | 16 | 3 |
| SNORD20 : 18S-1806 | 16 | 3 |
| SNORD35 : 28S-2791 | 16 | 2 |
| SNORD100 : 18S-436 | 13 | 1 |
| SNORD110 : 18S-1477 | 13 | 3 |
| SNORD110 : 18S-1573 | 13 | 3 |
| SNORD126 : 18S-84 | 13 | 1 |
| SNORD2 : 18S-1736 | 13 | 1 |
| snoU83B : 18S-1519 | 13 | 1 |
| SNORD103 : 18S-1668 | 12 | 1 |
| U8 : 18S-1210 | 12 | 1 |
| snoU83B : 28S-3659 | 12 | 1 |
| SNORD25 : 28S-44 | 11 | 1 |
| snoZ17 : 18S-409 | 10 | 2 |
| snoU83B : 28S-3641 | 10 | 1 |
| SNORD35 : 18S-441 | 9 | 1 |
| SNORD35 : 18S-856 | 9 | 4 |
| U3 : 28S-3031 | 9 | 3 |
| SNORD30 : 18S-1731 | 8 | 1 |
| SNORD33 : 18S-1326 | 8 | 3 |
| SNORD24 : 18S-22 | 8 | 1 |
| snoU83B : 18S-1199 | 8 | 1 |
| SNORD103 : 18S-1739 | 8 | 1 |
| SNORD11B : 18S-1605 | 7 | 2 |
| SNORD16 : 28S-2844 | 7 | 1 |
| SNORD35 : 18S-1667 | 7 | 1 |
| SNORD37 : 28S-3697 | 7 | 1 |
| SNORD48 : 18S-1231 | 7 | 2 |
| SNORD65 : 18S-157 | 7 | 1 |
| SNORD2 : 18S-130 | 6 | 1 |
| SNORD31 : 28S-2903 | 6 | 1 |
| SNORD43 : 18S-127 | 6 | 1 |
| SNORD69 : 18S-1285 | 6 | 1 |
| snoU83B : 18S-128 | 6 | 3 |
| SNORD100 : 28S-4576 | 5 | 1 |
| U8 : 18S-411 | 5 | 1 |
| U8 : 18S-412 | 5 | 1 |
| U8 : 18S-1605 | 5 | 1 |
| SNORD14 : 18S-1512 | 5 | 1 |
| SNORD33 : 18S-1328 | 5 | 1 |
| SNORD57 : 18S-99 | 5 | 1 |
| SNORD70 : 18S-79 | 5 | 1 |
| SNORD79 : 18S-114 | 5 | 1 |
| snoU83B : 18S-507 | 5 | 1 |
| snoU83B : 18S-599 | 5 | 1 |
| SNORD103 : 28S-2823 | 5 | 1 |
| SNORD142;ZL68 : 18S-1596 | 4 | 2 |
| SNORD36 : 28S-15 | 4 | 1 |
| SNORD38 : 18S-467 | 4 | 1 |
| SNORD38 : 18S-476 | 4 | 1 |
| SNORD44 : 18S-818 | 4 | 1 |
| SNORD44 : 18S-829 | 4 | 1 |
| snoZ17 : 18S-1574 | 4 | 1 |
| SNORD58 : 18S-1510 | 4 | 1 |
| SNORND104 : 18S-144 | 3 | 1 |
| SNORND104 : 5.8S-50 | 3 | 1 |
| SNORD110 : 18S-1674 | 3 | 2 |
| snoR38 : 18S-70 | 3 | 1 |
| SNORD27 : 28S-2347 | 3 | 1 |
| SNORD30 : 18S-417 | 3 | 1 |
| SNORD30 : 18S-433 | 3 | 1 |
| SNORD36 : 18S-1739 | 3 | 1 |
| SNORD42 : 18S-43 | 3 | 1 |
| SNORD45 : 18S-159 | 3 | 1 |
| SNORD58 : 18S-613 | 3 | 1 |
| SNORD59 : 18S-84 | 3 | 1 |
| snoZ40 : 18S-1571 | 3 | 1 |
| SNORD62 : 28S-2394 | 3 | 2 |
| SNORD63 : 18S-893 | 3 | 1 |
| SNORD66 : 28S-13 | 3 | 2 |
| snoMBII-202 : 18S-1511 | 3 | 1 |
| SNORA46 : 18S-649 | 2 | 1 |
| SNORD100 : 18S-405 | 2 | 1 |
| SNORD100 : 18S-411 | 2 | 1 |
| SNORND104 : 28S-1576 | 2 | 1 |
| snoU105B : 28S-57 | 2 | 1 |
| SNORD12 : 28S-2739 | 2 | 1 |
| snoU13 : 18S-1725 | 2 | 1 |
| SNORD133;ZL142 : 18S-1758 | 2 | 1 |
| SNORD135;ZL2 : 18S-110 | 2 | 1 |
| SNORD15 : 18S-27 | 2 | 1 |
| SNORD19 : 18S-702 | 2 | 1 |
| snoR38 : 28S-2871 | 2 | 1 |
| SNORD2 : 18S-1164 | 2 | 1 |
| SNORD31 : 18S-1172 | 2 | 1 |
| SNORD33 : 18S-1598 | 2 | 1 |
| SNORD34 : 18S-1485 | 2 | 1 |
| SNORD34 : 5.8S-14 | 2 | 1 |
| SNORD35 : 18S-47 | 2 | 2 |
| SNORD35 : 18S-828 | 2 | 1 |
| SNORD36 : 28S-3703 | 2 | 1 |
| SNORD36 : 18S-410 | 2 | 1 |
| SNORD36 : 18S-668 | 2 | 1 |
| SNORD38 : 28S-4979 | 2 | 2 |
| U3 : 28S-3446 | 2 | 2 |
| SNORD42 : 18S-461 | 2 | 1 |
| snoZ17 : 18S-121 | 2 | 1 |
| SNORD39 : 28S-2791 | 2 | 1 |
| SNORD59 : 18S-1616 | 2 | 1 |
| snoZ40 : 18S-1584 | 2 | 1 |
| SNORD65 : 18S-396 | 2 | 1 |
| SNORD65 : 18S-1261 | 2 | 1 |
| SNORD65 : 28S-1722 | 2 | 1 |
| SNORD83 : 18S-806 | 2 | 1 |
| SNORD86 : 18S-1219 | 2 | 2 |
| SNORD100 : 18S-1734 | 1 | 1 |
| SNORD100 : 18S-1738 | 1 | 1 |
| U8 : 18S-600 | 1 | 1 |
| SNORD12 : 18S-1536 | 1 | 1 |
| SNORD14 : 18S-462 | 1 | 1 |
| snoR38 : 18S-114 | 1 | 1 |
| snoR38 : 18S-1575 | 1 | 1 |
| snoR38 : 18S-43 | 1 | 1 |
| SNORD25 : 18S-1490 | 1 | 1 |
| SNORD30 : 18S-1383 | 1 | 1 |
| SNORD36 : 18S-205 | 1 | 1 |
| SNORD36 : 18S-1738 | 1 | 1 |
| SNORD45 : 18S-159 | 1 | 1 |
| SNORD65 : 18S-416 | 1 | 1 |
| snoMBII-202 : 18S-428 | 1 | 1 |
| SNORD73 : 28S-1747 | 1 | 1 |
| snoU83B : 18S-1199 | 1 | 1 |
| SNORD91 : 28S-4588 | 1 | 1 |
| SNORD53_SNORD92 : 18S-619 | 1 | 1 |

#

# Table S5: Blocking hybrid counts

| Interaction | Hybrid count | Experiment count |
| --- | --- | --- |
| snoZ17 : 28S-4589 | 9 | 1 |
| snoZ17 : 28S-4588 | 8 | 1 |
| SNORD91 : 18S-1052 | 8 | 2 |
| snoZ17 : 28S-4592 | 4 | 1 |
| snoZ17 : 28S-4593 | 4 | 1 |
| SCARNA17 : 18S-93 | 3 | 1 |
| SNORD33 : 18S-93 | 1 | 1 |
| SNORD91 : 18S-1056 | 4 | 1 |
| snoZ17 : 28S-4596 | 3 | 1 |
| snoZ17 : 28S-4598 | 3 | 1 |
| SCARNA17 : 18S-95 | 2 | 1 |
| SNORD33 : 18S-95 | 1 | 1 |
| SNORD34 : 18S-22 | 2 | 1 |
| SNORD34 : 18S-27 | 2 | 1 |
| SNORD34 : 18S-28 | 2 | 1 |
| SCARNA17 : 18S-90 | 1 | 1 |
| SNORD33 : 18S-90 | 1 | 1 |
| snoZ17 : 28S-4590 | 1 | 1 |
| snoZ17 : 28S-4603 | 1 | 1 |
| SNORD34 : 18S-21 | 1 | 1 |
| SNORD33 : 18S-84 | 1 | 1 |

#

# Table S6: Structural hybrid counts

| Interaction | Hybrid count | Experiment count |
| --- | --- | --- |
| U3 | 6,827 | 14 |
| U8 | 2,118 | 12 |
| snoU83B | 1,124 | 11 |
| SNORD58 | 1,022 | 3 |
| SNORD14 | 834 | 11 |
| snoU13 | 812 | 13 |
| SNORD62 | 580 | 7 |
| SNORD38 | 543 | 6 |
| SNORD91 | 501 | 4 |
| snoR38 | 472 | 4 |
| SNORD103 | 429 | 4 |
| SNORD44 | 419 | 1 |
| SNORD83 | 416 | 5 |
| SNORD107 | 396 | 2 |
| SNORD100 | 394 | 7 |
| SNORD36 | 380 | 3 |
| SNORD110 | 342 | 7 |
| SNORD34 | 321 | 3 |
| SNORD2 | 310 | 2 |
| snoZ17 | 307 | 3 |
| SNORD16 | 301 | 6 |
| SNORD45 | 295 | 3 |
| snoMBII-202 | 294 | 6 |
| SNORD31 | 255 | 5 |
| snoU2_19 | 251 | 5 |
| others | 8,057 | 16 |

# Table S7: snoRNA interactions overlapping mRNA methylation sites

HEK293

| overlap count | | mRNA | snoRNA | | |
| --- | --- | --- | --- | --- | --- |
| 6 | ENSG00000123416:::TUBA1B:::mRNA | | | snoID0146:::SNORD85:::snoRNA |  |
| 6 | ENSG00000255823:::MTRNR2L8:::mRNA | | | snoID0273:::SNORD3A:::snoRNA |  |
| 4 | ENSG00000142937:::RPS8:::mRNA | | | snoID0146:::SNORD85:::snoRNA |  |
| 4 | ENSG00000156508:::EEF1A1:::mRNA | | | snoID0273:::SNORD3A:::snoRNA |  |
| 4 | ENSG00000231500:::RPS18:::mRNA | | | snoID0273:::SNORD3A:::snoRNA |  |
| 3 | ENSG00000105193:::RPS16:::mRNA | | | ENSG00000200496:::U8:::snoRNA |  |
| 3 | ENSG00000140988:::RPS2:::mRNA | | | snoID0273:::SNORD3A:::snoRNA |  |
| 3 | ENSG00000142534:::RPS11:::mRNA | | | snoID0273:::SNORD3A:::snoRNA |  |
| 3 | ENSG00000255823:::MTRNR2L8:::mRNA | | | snoID0043:::SNORD58C:::snoRNA |  |
| 2 | ENSG00000105372:::RPS19:::mRNA | | | snoID0084:::SNORD69:::snoRNA |  |
| 2 | ENSG00000111640:::GAPDH:::mRNA | | | snoID0062:::SNORD16:::snoRNA |  |
| 2 | ENSG00000142937:::RPS8:::mRNA | | | snoID0465:::SNORA34:::snoRNA |  |
| 2 | ENSG00000167526:::RPL13:::mRNA | | | snoID0080:::SNORD66:::snoRNA |  |
| 2 | ENSG00000228716:::DHFR:::mRNA | | | snoID0043:::SNORD58C:::snoRNA |  |
| 2 | ENSG00000255823:::MTRNR2L8:::mRNA | | | snoID0010:::SNORD83A:::snoRNA |  |
| 2 | ENSG00000255823:::MTRNR2L8:::mRNA | | | snoID0108:::SNORD62B:::snoRNA |  |
| 2 | ENSG00000263809:::KRBA2:::mRNA | | | snoID0114:::SNORD83B:::snoRNA |  |
| 1 | ENSG00000111669:::TPI1:::mRNA | | | snoID0002:::SNORD38A:::snoRNA |  |
| 1 | ENSG00000115268:::RPS15:::mRNA | | | snoID0273:::SNORD3A:::snoRNA |  |
| 1 | ENSG00000147403:::RPL10:::mRNA | | | snoID0273:::SNORD3A:::snoRNA |  |
| 1 | ENSG00000231500:::RPS18:::mRNA | | | snoID0171:::SNORD58B:::snoRNA |  |
| 1 | ENSG00000233927:::RPS28:::mRNA | | | snoID0273:::SNORD3A:::snoRNA |  |
| 1 | ENSG00000255823:::MTRNR2L8:::mRNA | | | snoID0015:::SNORD50A:::snoRNA |  |

HELA

| Overlap count | mRNA | snoRNA |
| --- | --- | --- |
| 9 | ENSG00000198034:::RPS4X:::mRNA | snoID0273:::SNORD3A:::snoRNA |
| 5 | ENSG00000147403:::RPL10:::mRNA | snoID0273:::SNORD3A:::snoRNA |
| 4 | ENSG00000123416:::TUBA1B:::mRNA | snoID0146:::SNORD85:::snoRNA |
| 4 | ENSG00000147403:::RPL10:::mRNA | snoID0114:::SNORD83B:::snoRNA |
| 4 | ENSG00000147403:::RPL10:::mRNA | snoID0155:::SNORD34:::snoRNA |
| 4 | ENSG00000156508:::EEF1A1:::mRNA | snoID0273:::SNORD3A:::snoRNA |
| 4 | ENSG00000174748:::RPL15:::mRNA | snoID0155:::SNORD34:::snoRNA |
| 4 | ENSG00000256618:::MTRNR2L1:::mRNA | snoID0180:::SNORD42A:::snoRNA |
| 3 | ENSG00000174748:::RPL15:::mRNA | snoID0273:::SNORD3A:::snoRNA |
| 2 | ENSG00000126267:::COX6B1:::mRNA | snoID0273:::SNORD3A:::snoRNA |
| 2 | ENSG00000142937:::RPS8:::mRNA | snoID0146:::SNORD85:::snoRNA |
| 2 | ENSG00000150636:::CCDC102B:::mRNA | snoID0010:::SNORD83A:::snoRNA |
| 2 | ENSG00000150636:::CCDC102B:::mRNA | snoID0011:::SNORD10:::snoRNA |
| 2 | ENSG00000150636:::CCDC102B:::mRNA | snoID0166:::SNORD57:::snoRNA |
| 2 | ENSG00000167526:::RPL13:::mRNA | snoID0080:::SNORD66:::snoRNA |
| 2 | ENSG00000231500:::RPS18:::mRNA | snoID0273:::SNORD3A:::snoRNA |
| 1 | ENSG00000115268:::RPS15:::mRNA | snoID0273:::SNORD3A:::snoRNA |
| 1 | ENSG00000140988:::RPS2:::mRNA | snoID0273:::SNORD3A:::snoRNA |
| 1 | ENSG00000140988:::RPS2:::mRNA | snoID0273:::SNORD3A:::snoRNA |

______________________________________________________________________________________
