# Supplementary material for: Systematic mapping of small nucleolar RNA interactions in human cells
Source: RNA Biol. 2025 Nov 14;22(1):1–22. doi: 10.1080/15476286.2025.2589573 (PMC12645875; doi:10.1080/15476286.2025.2589573)
Supplement: Dunn Davis et al. Supp. Figs S1 - S13.docx [file KRNB_A_2589573_SM0067.docx]

**Dunn Davis et al. SUPPLEMENTARY MATERIALS**

**Dataset 1: All interactions recovered**

**Supplementary Figure Legends**

**Figure S1. Expression of tagged protein constructs**

Expression of HTP-tagged fibrillarin, NOP56 and NOP58 was assessed by western blotting. The endogenous (lower bands) and tagged fusion proteins (upper bands) were detected with anti-fibrillarin, anti-NOP56 and anti-NOP58, respectively. Insertion of non-tagged pcDNA5, was the negative control. The tags were also visualized with anti-FLAG.

**Figure S2. Single hits by biotype**

(A) Breakdown of total uncollapsed single hit counts for different RNA biotypes. (B) Breakdown of uncollapsed single hit counts for different RNA biotypes in Fibrillarin CLASH and FLASH experiments. (C) Breakdown of uncollapsed single hit counts for different RNA biotypes in NOP56 CLASH and FLASH experiments. (D) Breakdown of uncollapsed single hit counts for different RNA biotypes in Fibrillarin FLASH experiments.

**Figure S3. Hybrids recovered by biotype**

(A) Breakdown of hybrid counts in snoRNA hybrids by RNA biotype. (B) Breakdown of counts of reproducible, stable snoRNA hybrids (with predicted folding energy of -12dG or below) by RNA biotype.

**Figure S4. Classes of non-methylating rRNA interactions**

(A, B) Breakdown of snoRNA-pre-47S hybrids involved in ancillary interactions and blocking

interactions respectively. The X axis labels show the snoRNA families involved, along with the co-ordinate of the relevant methylation site on the pre-47S sequence. (C) Breakdown of

snoRNA-pre-47S hybrids involved in structural interactions. The X axis labels show the relevant snoRNA species.

**Figure S5. Characterization of snoRNA-snoRNA interactions**

(A) Breakdown of reproducible intermolecular snoRNA-snoRNA hybrids by hybrid type. The hybrid type is obtained by joining the species name of each of the interacting snoRNAs. The order of the snoRNAs is not considered. (B) Breakdown of snoRNA-snoRNA hybrids that pass the filtering criteria described in figure 2A. The X axis labels show the snoRNAs and co-ordinate at which methylation is predicted to occur, and the snoRNA box 5 base pairs downstream of the methylating nucleotide. (C) Counts of snoRNA-snoRNA hybrids predicted to guide methylation with high confidence.

**Figure S6. Distribution of length and stability of snoRNA-mRNA hybrids**

(A) Histogram of fragment lengths in all hybrids. (B) Histogram of predicted folding energies in all hybrids. (C) Histogram of fragment lengths in reproducible, stable filtered snoRNA-mRNA hybrids with U3 and rRNA filters applied. (D) Histogram of predicted folding energies in reproducible, stable snoRNA-mRNA hybrids with U3 and rRNA filters applied.

**Figure S7. Conservation scores of snoRNA-mRNA interactions in human cells**

(A) Conservation relative to flanking regions for mRNA fragments of snoRNA-mRNA hybrids involving U3, and non-U3 C/D box snoRNAs, respectively. Beneath each conservation plot is a bar chart showing the distribution of the mRNA fragments of the relevant hybrids between branch point regions of the host gene (host bp), non-branch point regions of the host gene (host non-bp), branch point regions of other genes (other bp) and non-branch point regions of other genes (other non-bp). (B) Conservation relative to flanking regions, split by proportion of hybrids conserved. These plots show that for the majority of interactions and hybrids, there is a clear peak in conservation corresponding to the location of the mRNA fragment of the hybrid. However, there is a small number of interactions with many hybrids that show a large decrease in conservation relative to flanks. This obscures the general pattern of conservation in the overall chart. (C) Three snoRNA-EIF4A2 interactions that show a drop in conservation relative to flanking regions. The three most common interactions with reduced conservation each involves an intron branch point region in EIF4A2. (D) Genome browser track showing the location of the mRNA fragment of SNORD2-EIF4A2 hybrids. In this case, the upstream flanking region overlaps the SNORD2 gene, and the downstream flanking region overlaps an exon of EIF4A2, both of which are highly conserved relative to the intron branch point region bound by the mRNA fragment of the hybrids, explaining the dip in conservation.

**Figure S8. Potential methylating interactions with mRNAs**

(A) Counts of filtered snoRNA-mRNA hybrids meeting the criteria for classification as high confidence methylating hybrids, broken down by interaction. (B) Counts of filtered snoRNA mRNA hybrids meeting the criteria for classification as potentially methylating hybrids, broken down by interaction.

**Figure S9. GO term enrichment analysis of mRNAs with reproducible snoRNA hybrids**

The graph shows the number of mRNAs as fraction of the total number of genes included under the GO term. Spot sizes indicate the absolute number of mRNAs recovered and the color reflects the statistical significance of the enrichment (p_adjusted).

**Figure S10. Filtering steps applied to hybrids for U3**

(A) Schematic representation of the filtering steps applied to different classes of RNA-RNA hybrids. Initial, single hit filtering was applied to all hybrids to filter out miscalled single reads. Filtering for reproducibility and stability was then applied to all hybrids. Additional U3 filtering was applied to all hybrids between snoRNAs and other biotypes to filter out miscalled U3-U3 intermolecular hybrids. Finally, additional rRNA filtering was applied to all hybrids between snoRNAs and other non-rRNA biotypes, to filter out miscalled snoRNA-rRNA hybrids. (B) As an example, the profile plot shows the distribution of filtered, reproducible SNORD3A (U3) hybrids across SNORD3A by biotype. Each line shows the profile for a different SNORD3A associated RNA biotype. The grey shaded regions represent 20 base pair flanking regions upstream and downstream of SNORD3A. The light blue shaded region represents the D box of SNORD3A, the purple line shows the nucleotide 5 base pairs upstream of the D box of SNORD3A, and the light green shaded region represents the D' box of SNORD3A.

**Figure S11. Validation of U3 depletion**

Total RNA was extracted following depletion of U3 for 3 days and separated on a denaturing 8% polyacrylamide gel containing urea. Mock depletion without ASO and control depletion of GFP are also shown, in four biological replicates. (A) Total low molecular weight RNA stained with EthBr. (B) Northern analysis of the U3 and U8 RNA snoRNAs, and the 7SL RNA component of the signal recognition particle (SRP) -used as loading control. Residual levels of U3 expression relative to the corresponding mock are indicated. Samples shown in lanes 1, 3, 4, 6, 7 and 9 were used for RNA sequencing. ASO sequences are reported in (Langhendries et al., 2016).

**Figure S12. Changes in mRNA abundance following U3 depletion**

(A) Volcano plot showing the changes in mRNA abundance following 3 days of U3 depletion, compared to a mock control, with U3 interactions found in filtered U3-mRNA hybrids highlighted in dark green. (B) Volcano plot showing the changes in mRNA abundance following 2 days of U3 depletion, compared to a mock control, with U3 interactions found in filtered U3-mRNA hybrids highlighted in dark green.

**Figure S13. GO term enrichment analyses for mRNAs with altered abundance following U3 depletion.**

The graphs show the number of mRNAs as fraction of the total number of genes included under the GO term. Spot sizes indicate the absolute number of mRNAs recovered and the color reflects the statistical significance of the enrichment (p_adjusted).


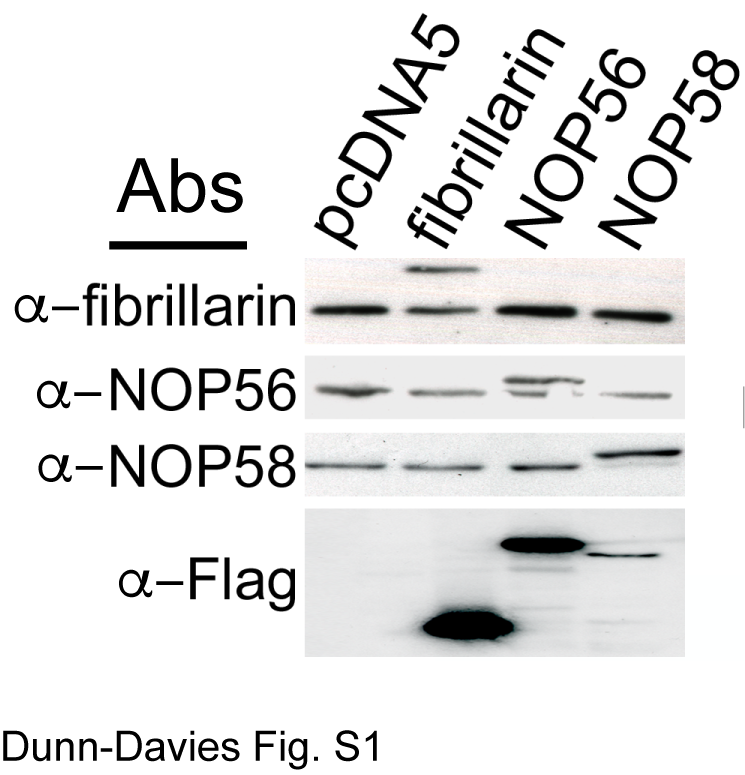


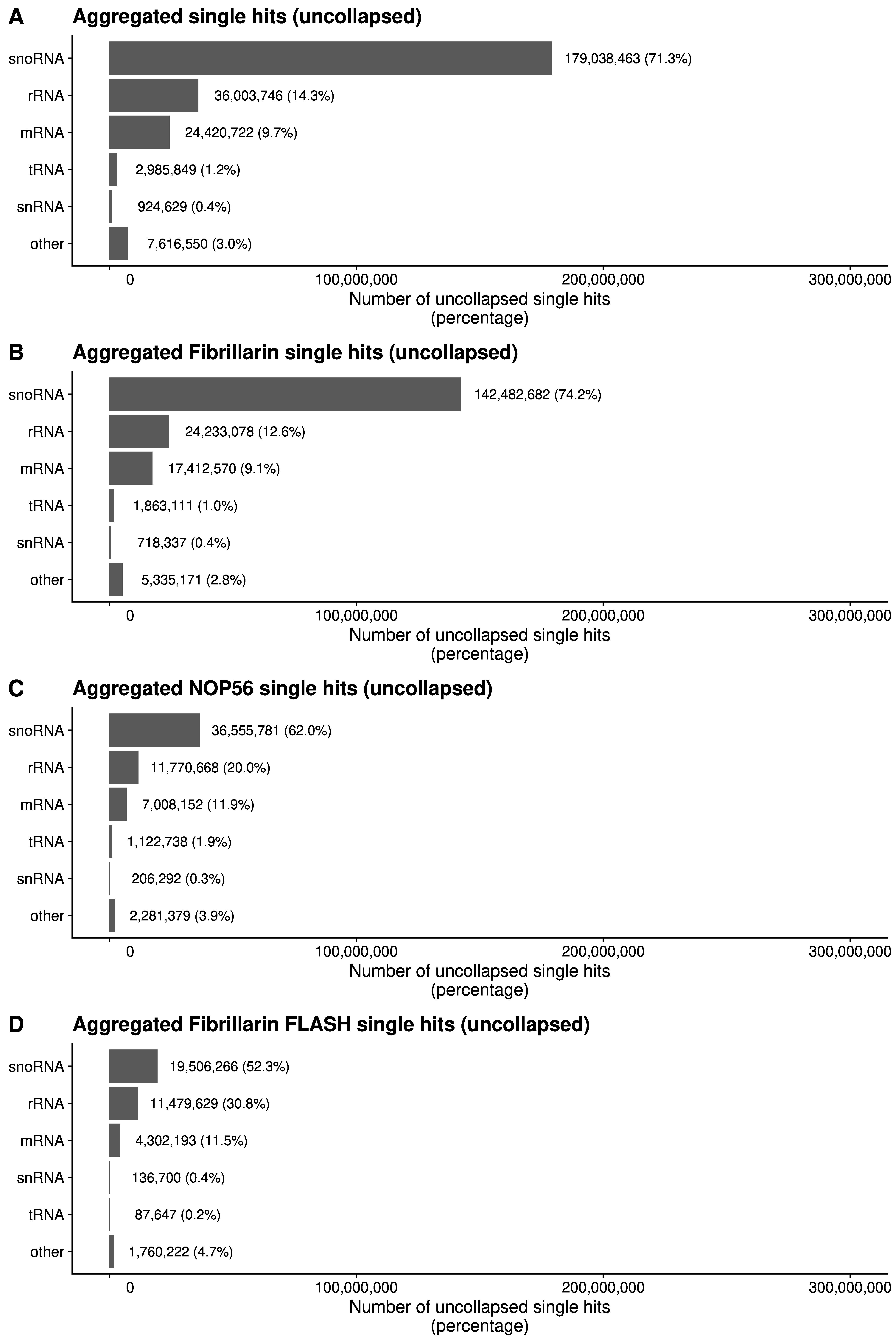
Dunn Davies Fig. S2

Dunn Davies Fig. S
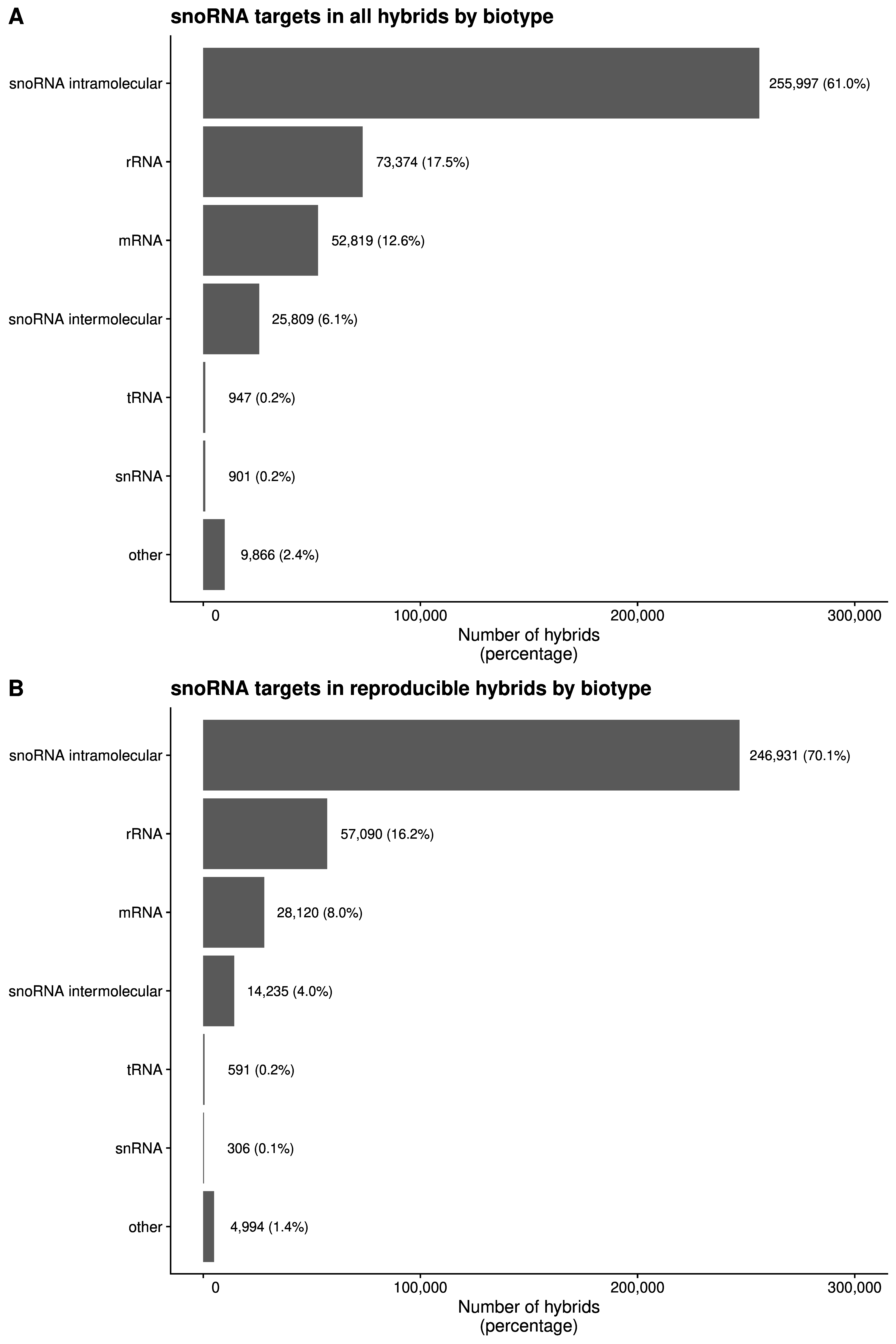
3

Dunn Davies Fig. S
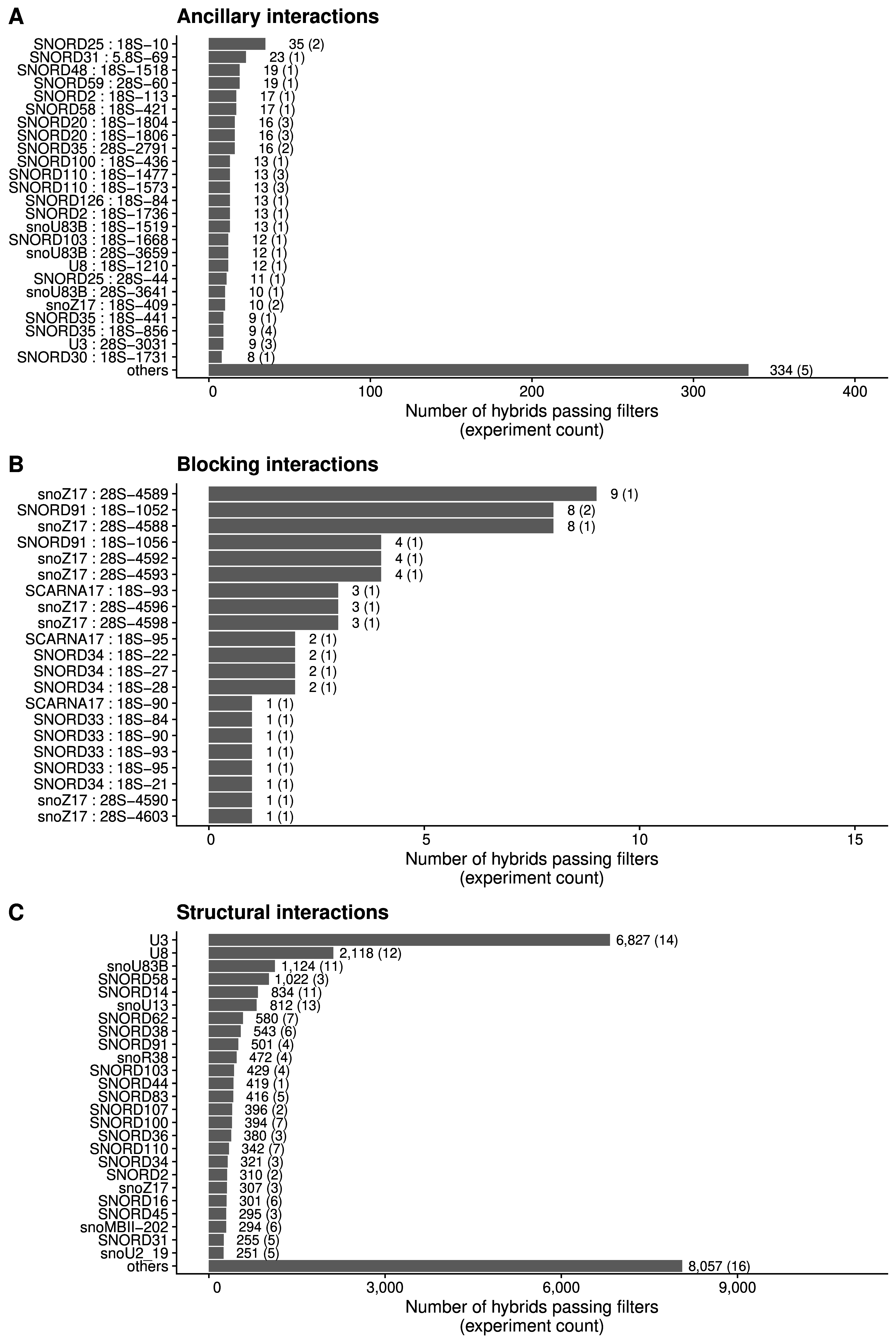
4

Dunn Davies Fig. S
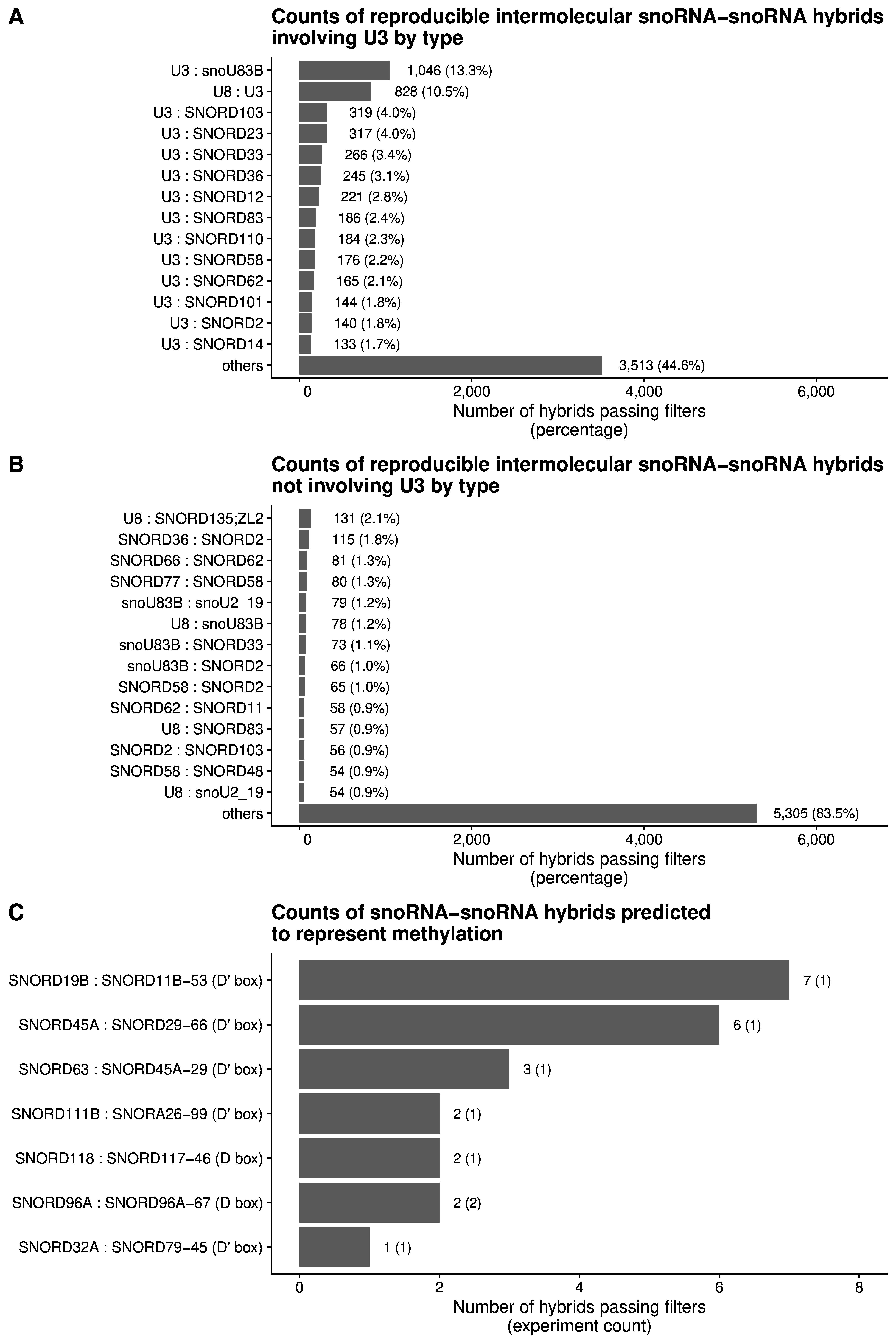
5

Dunn Davies Fig. S
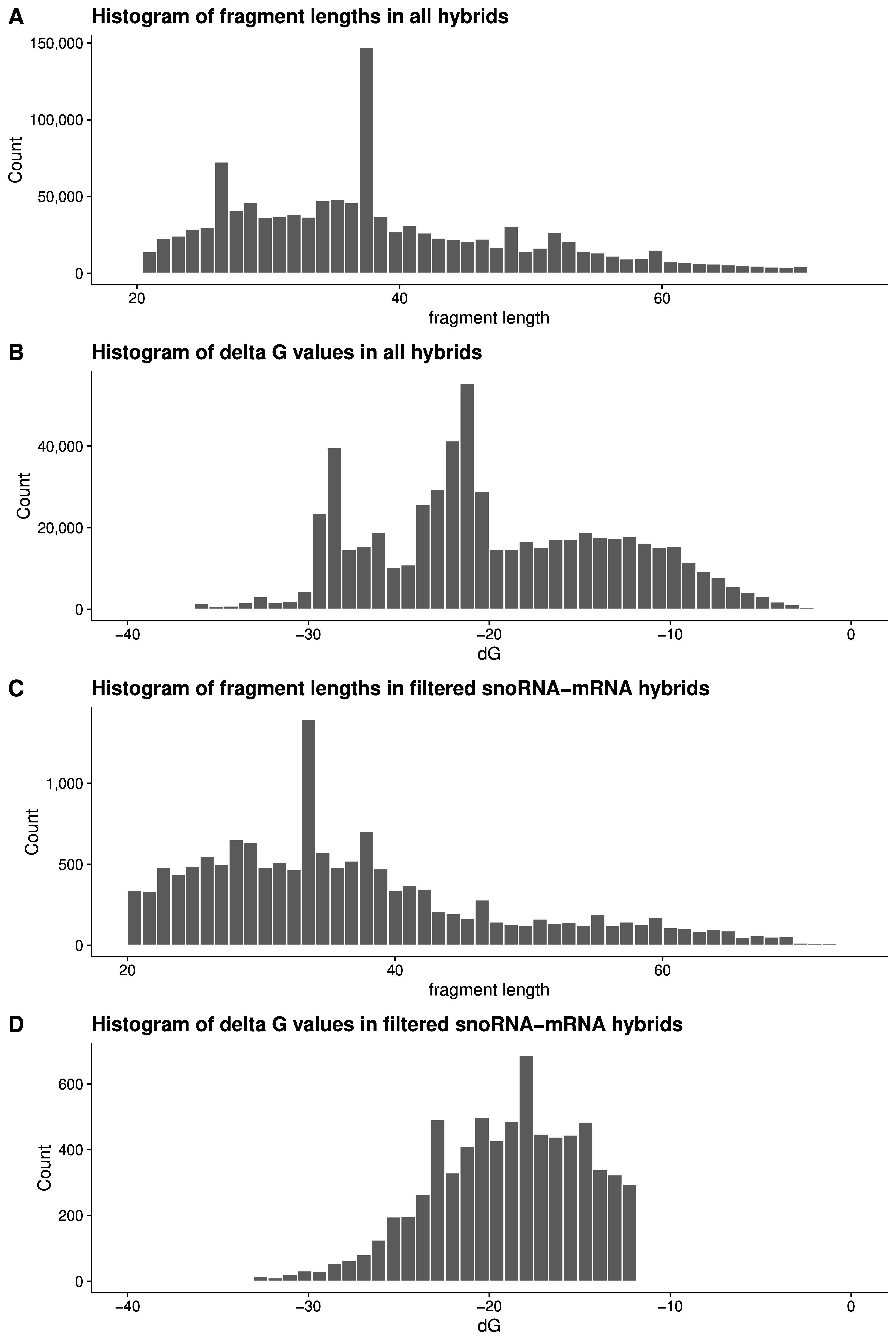
6

Dunn Davies Fig. S

7



Dunn Davies Fig. S8

Dunn Davies Fig. S9
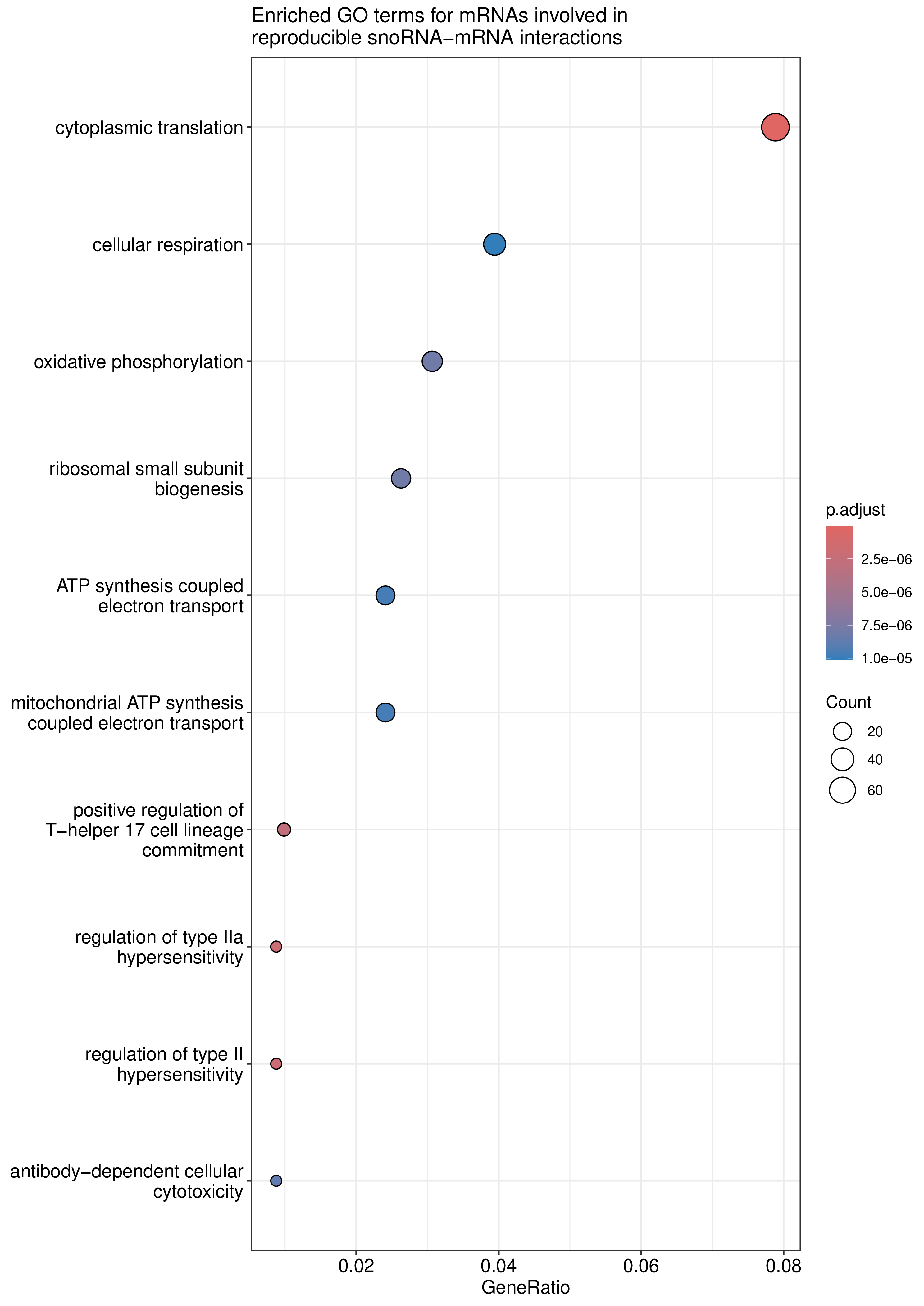


Dunn Davies Fig. S
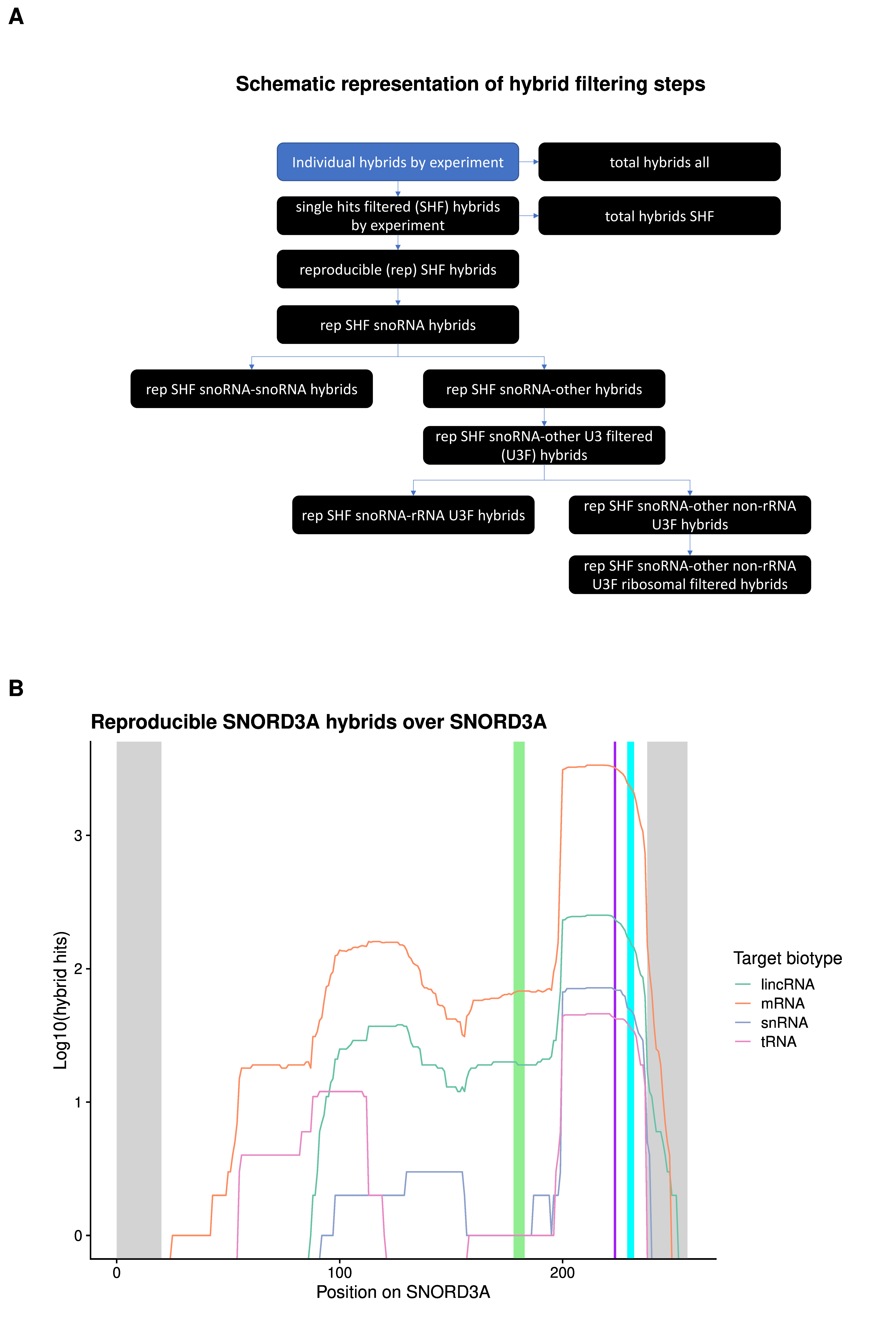
10


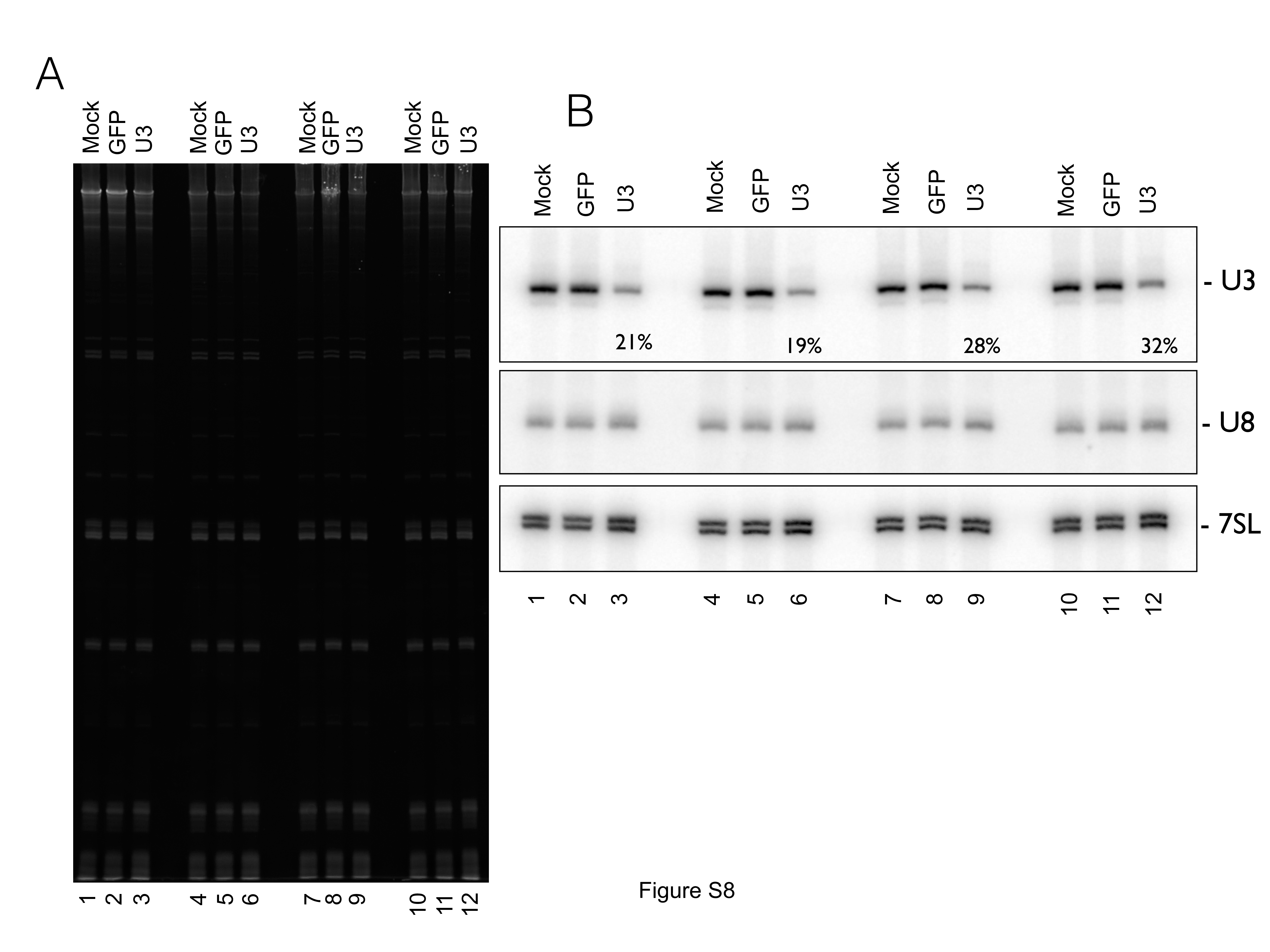


Dunn Davies Fig. S11

Dunn Davies Fig. S
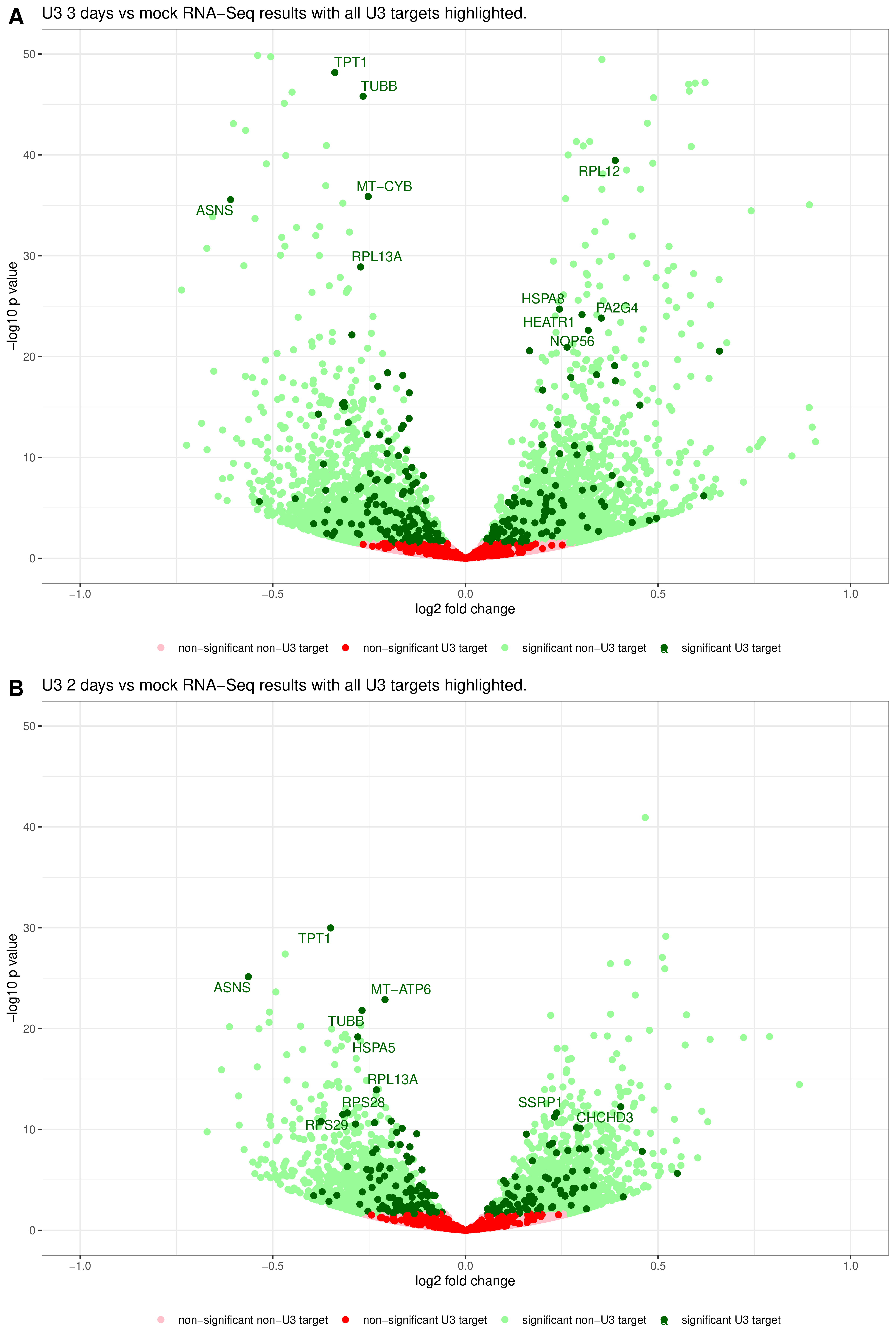
12


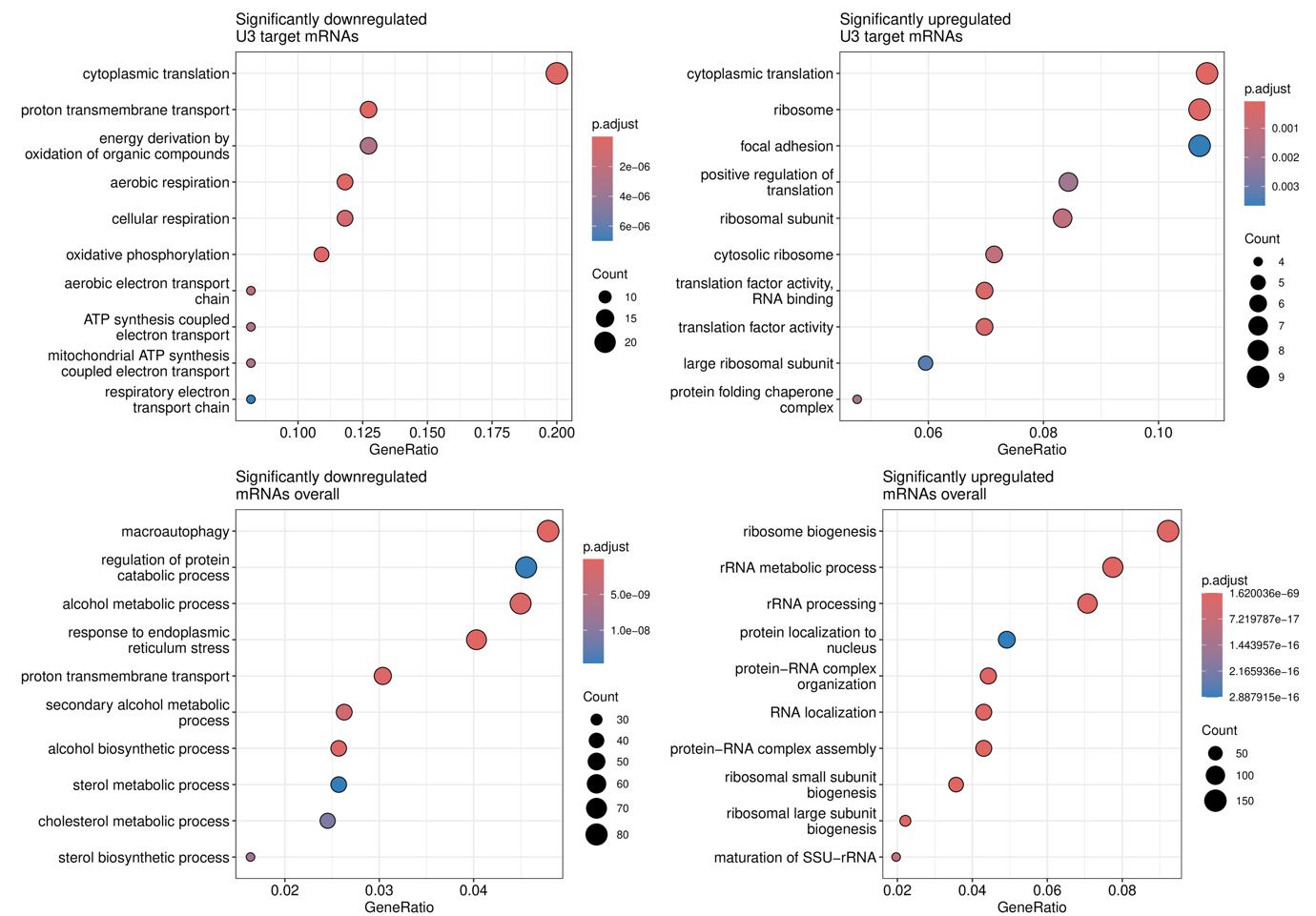


Dunn Davies Fig. S13
